# Anti-Fouling Effects of Saponin-Containing Crude Extracts from Tropical Indo-Pacific Sea Cucumbers

**DOI:** 10.3390/md18040181

**Published:** 2020-03-31

**Authors:** Elham Kamyab, Norman Goebeler, Matthias Y. Kellermann, Sven Rohde, Miriam Reverter, Maren Striebel, Peter J. Schupp

**Affiliations:** 1 Institute for Chemistry and Biology of the Marine Environment (ICBM), Carl-von-Ossietzky University Oldenburg, Schleusenstrasse 1, 26382 Wilhelmshaven, Germany; norman.gobeler@helsinki.fi (N.G.); matthias.kellermann@uol.de (M.Y.K.); sven.rohde@uol.de (S.R.); miriam.reverter@uol.de (M.R.); maren.striebel@uol.de (M.S.); 2 Tvärminne Zoological Station, University of Helsinki, J.A. Palmènin tie 260, 10900 Hanko, Finland; 3 Helmholtz Institute for Functional Marine Biodiversity at the University of Oldenburg (HIFMB), Ammerländer Heerstrasse 231, D-26129 Oldenburg, Germany

**Keywords:** holothurian, diatom, anti-fouling compounds, marine natural products, saponins, triterpene glycosides, mass spectrometry

## Abstract

Sea cucumbers are bottom dwelling invertebrates, which are mostly found on subtropical and tropical sea grass beds, sandy reef flats, or reef slopes. Although constantly exposed to fouling communities in these habitats, many species are surprisingly free of invertebrate epibionts and microfouling algae such as diatoms. In our study, we investigated the anti-fouling (AF) activities of different crude extracts of tropical Indo-Pacific sea cucumber species against the fouling diatom *Cylindrotheca closterium*. Nine sea cucumber species from three genera (i.e., *Holothuria*, *Bohadschia*, *Actinopyga*) were selected and extracted to assess their AF activities. To verify whether the sea cucumber characteristic triterpene glycosides were responsible for the observed potent AF activities, we tested purified fractions enriched in saponins isolated from *Bohadschia argus*, representing one of the most active anti-fouling extracts. Saponins were quantified by vanillin-sulfuric acid colorimetric assays and identified by LC-MS and LC-MS/MS analyses. We were able to demonstrate that AF activities in sea cucumber extracts were species-specific, and growth inhibition as well as attachment of the diatom to surfaces is dependent on the saponin concentration (i.e., *Actinopyga* contained the highest quantities), as well as on the molecular composition and structure of the present saponins (i.e., *Bivittoside D* derivative was the most bioactive compound). In conclusion, the here performed AF assay represents a promising and fast method for selecting the most promising bioactive organism as well as for identifying novel compounds with potent AF activities for the discovery of potentially novel pharmacologically active natural products.

## 1. Introduction

Biofouling is the colonization process of micro- (i.e., protozoa, bacteria, fungi and diatoms) or macro-organisms (i.e., algae and invertebrates) on either living (known as epibiosis) or artificial substrates [1,2]. Mckenzie and Grigolava (1996) described that epibiosis decreased host fitness, their survival rate, and species abundance, as well as affects their community composition [3]. Fouling on living surfaces of marine invertebrates can increase their friction as well as their body weight, and thus reduces speed, elasticity, and flexibility of the fouled organism, which in turn may lead to reduced viability and death [4,5]. Shading by fouling organisms can also impact negatively on the growth rate of the fouled organisms due to a reduced photosynthetic rate of macroalgae [6]. Biofouling processes are however not only relevant from an ecological perspective, but they have also important economic implications [4]. Marine biofouling shortens the lifespan and increases the maintenance costs of underwater constructions like ship hulls and aquaculture cages [7,8]. It also increases the weight and the friction of a ship, which in turn decreases the maximum cruising speed as well as increases fuel consumption [7,9]. In order to counteract the biofilm production on these structures, various synthetic anti-fouling (AF) paints that contain toxicants such as mercury (mercuric oxide (HgO), mercuric arsenate (AsO_4_Hg_3_)), arsenic (arsenic trioxide (As_2_O_3_)), copper (cuprous arsenite (AsO_3_Cu_3_)), as well as organotins (mainly tributyltin (TBT) based compounds) and rosin-based paint, have been applied in the past [7,9]. However, there are numerous studies showing that all the latter paints are hazardous for the environment and negatively affect the growth rate and reproduction of both fouling and non-fouling marine organisms [7,8,10,11,12]. As a result of such studies, production and application of TBT-based AF paints was internationally banned in the 1990s [7,13], and substituted with copper-based and booster biocides. However, recent research showed that these compounds still display toxic effects on marine organisms. The use of natural products as biological-based AF biocides in coating has been suggested as a new sustainable alternative, since they generally show biocompatibility, biodegradability, and thus their toxicity effects (if any) will not accumulate and generate long-lasting perturbations in the environment [14,15,16,17,18]. Engineering of an effective biological-based AF coating may not only protect the marine environment, but could also have substantial economic benefits by increasing the lifespan of underwater structures and by reducing the fuel consumption rate in the shipping industry (i.e., 60$ billion per annum). Furthermore, reduced fuel consumption also decreases carbon dioxide and sulfur dioxide emissions to the atmosphere [19,20,21] and therefore mitigate the effects of world-wide shipping to climate change.

**Figure 1:**
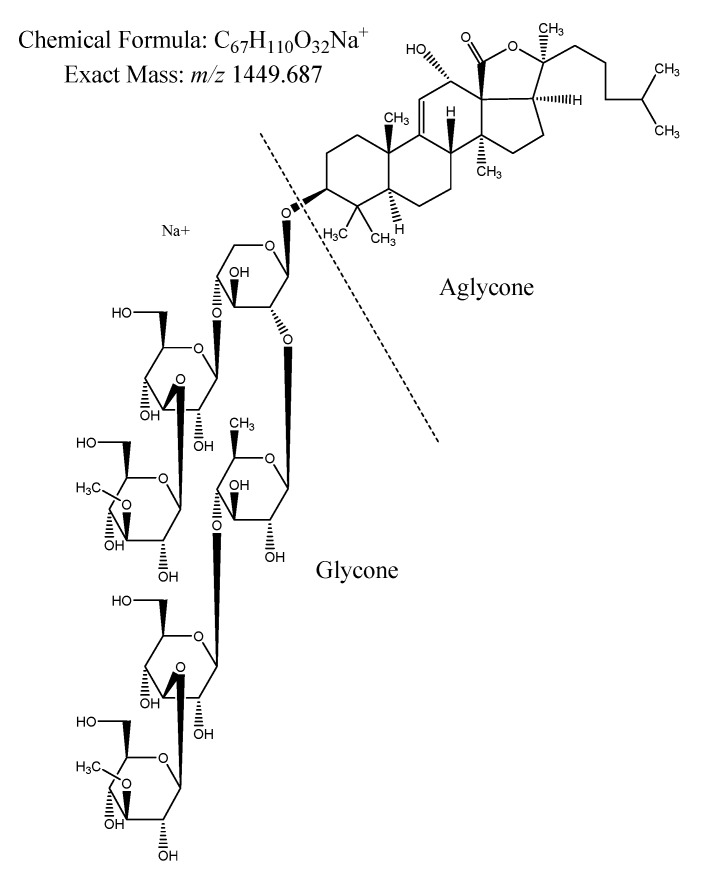
Structure of the saponin molecule “*bivittoside D*”, *m/z* 1449.687 [M + Na]^+^ [56], consisting of the glycone and aglycone moieties (produced with ChemDraw, version 16.0.1.4 (77)).

**Figure 2:**
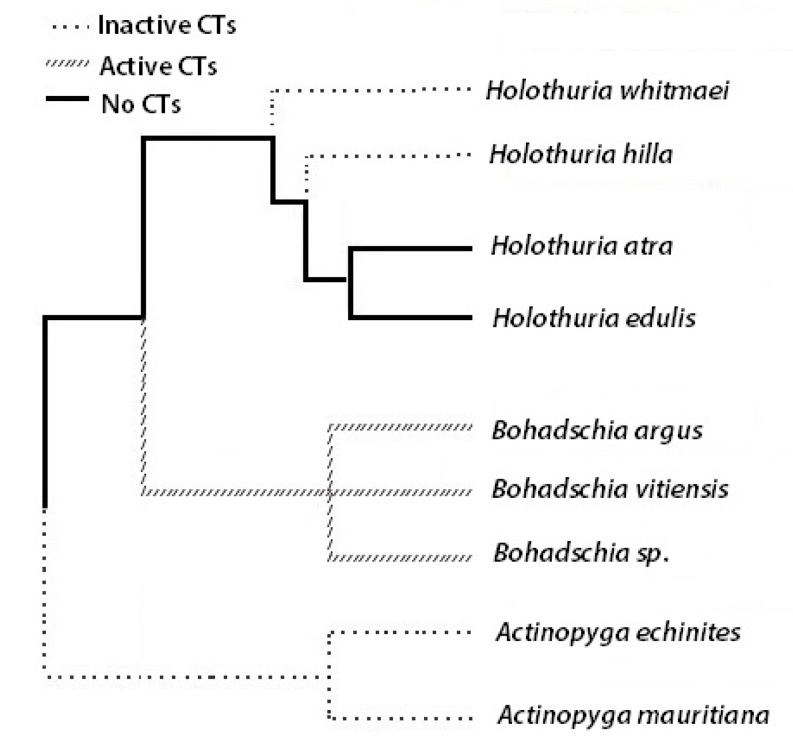
Phylogeny tree of the here studied sea cucumbers (CT = cuvierian tubule; adapted from [57,58,59,60].

In order to avoid the negative effects from unwanted epibioses, organisms can either accept and tolerate the presence of the fouling organisms (i.e., by developing a symbiosis) or avoid them, by either changing their habitat or developing chemical defenses [3,22,23,24]. AF defenses of marine organisms include mucus secretion (e.g., in sea star *Marthasterias glacialis*; [25]), shedding, microroughness, burrowing, scraping, and cleaning their body wall as well as chemical defense [4,26,27,28]. Chemical anti-fouling compounds can have various modes of action. They can be toxic to epibionts [29,30,31], inhibit settlement of larvae from fouling organisms [5,32,33] or prevent development of bacterial biofilms by disrupting bacterial communication via inhibition of the bacterial acylated homoserine lactone (AHL) signaling pathways [34]. Until 2017, almost 200 different AF compounds were described from marine invertebrates such as echinoderms, sponges, gorgonians and soft corals [35]. These AF compounds belong to various groups of terpenoids (i.e., triterpenes, sesquiterpenes and diterpenes), alkaloids, steroids, triterpene glycosides (saponins), polyacetylenes, butenolides, peptides and phenol derivatives [30,35,36]. More recently, the AF activities of compounds isolated from sea cucumbers such as *Holothuria atra* [37,38], *H. nobilis* [38], *H. edulis* [39], *H. glaberrima* [40], *H. tubulosa* and *H. polii* [41] were reported, as these sea cucumbers keep their body surfaces conspicuously free of fouling organisms [3]. Echinoderms, and especially sea cucumbers, are known to produce a wide variety of triterpene glycosides or saponins [42]. Saponins are composed of a hydrophilic glycone and a hydrophobic aglycone (i.e., sapogenin; Figure 1) that, depending on the holothuroids (*cf.* Figure 2), are located in the Cuverian Tubules (CT), in its body wall and its viscera [43]. Because of the membranolytic activities of saponin, a wide range of bioactivities such as anti-bacterial, anti-fungal, anti-viral, anti-inflammatory, ichthyotoxic, as well as anti-fouling properties have been reported [42,44,45].

The colonization process of fouling organisms starts after the first contact of the respective surface to sea water [4]. After “biochemical conditioning,” which is initiated by adsorption of macromolecules to the surface, a bacterial biofilm develops. This is followed by the colonization of unicellular eukaryotes and algae such as diatoms [4]. One such algae is the meroplanktonic diatom *Cylindrotheca closterium*, that showed rapid growth particularly on surfaces [46,47]. This diatom species is also known to produce different types of hydrophilic and carbohydrate-rich extracellular polymeric substances (EPS) that often represent the major component of the extracellular aggregative matrix [46]. EPS plays a crucial role in the biofilm formation, and the microbial and physicochemical defenses of the diatom [48,49], their motility [50], cell to cell and cell to substratum adhesion [51], as well as in the settlement success and post larval growth of other organisms [52,53,54]. Thus, *C. closterium* has been used as a model organism in the past for early stage fouling studies [50,52,55]. 

The aim of this study is to determine AF activities of different crude extracts of tropical Indo-Pacific sea cucumber species against the fouling diatom species *C. closterium*. To identify phylogenetic differences in AF activities of sea cucumber species, we choose nine species from three different genera (i.e., *Holothuria*, *Bohadschia*, *Actinopyga*). Also, we tested purified fractions enriched in saponins as well as pure saponin compounds to verify whether these sea cucumber characteristic compounds were involved in the observed AF activities of sea cucumbers. 

## 2. Results

### 2.1. Anti-Fouling Effects of the Crude Extracts

Antifouling activity of the sea cucumber crude extracts was assessed by measuring biomass and attachment of the diatom *C. closterium*. To assess suspended algal biomass, chlorophyll a *(Chl a)* was extracted from the water samples, while *Chl a* content of diatoms attached to the substrate was used to evaluate diatom attachment. *Chl a* measurements are well established as a proxies for monitoring water quality, assessing phytoplankton biomass, and estimating primary production [61,62,63], while fluorometric measurements of *Chl a* concentrations are an efficient proxy to monitor the total biomass of diatoms in the water column and on the substrate. To determine the anti-fouling effects of the holothurian’s crude extracts, a logarithmic response ratio (LRR; see Section 4.1.5) of measured *Chl a* concentrations was calculated. Negative LRR reveals an anti-fouling effect of the extract with less *Chl a* in the treated compared to the control samples, while a positive LRR indicates a higher *Chl a* concentration and thus an increase in algal growth in the treatments compared to the control samples. 

**Figure 3:**
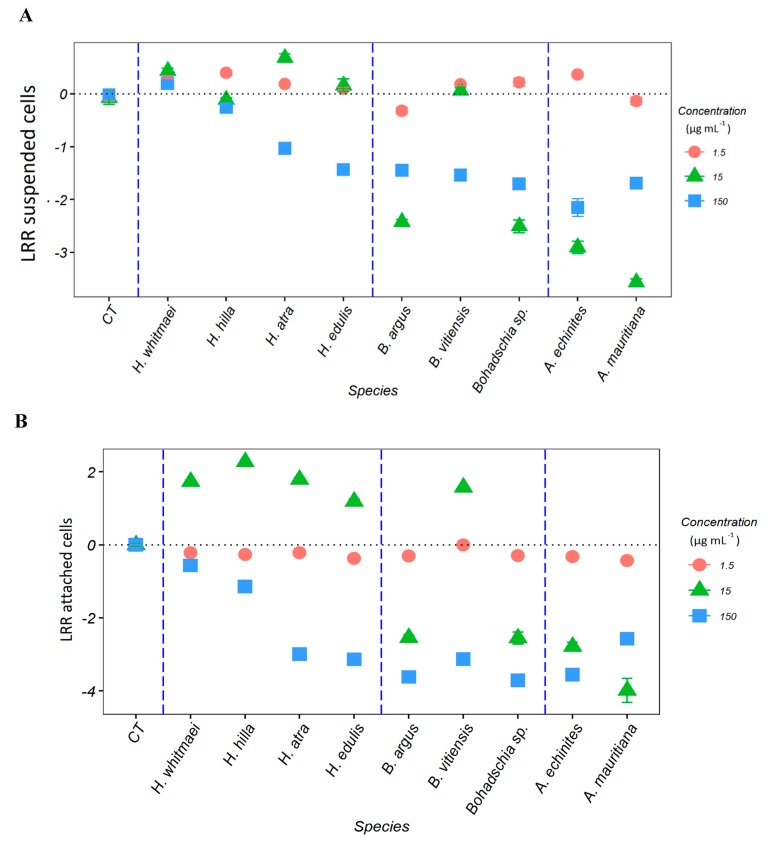
Logarithmic response ratio (LRR) of *C. closterium* after exposure to three different concentrations (150, 15 and 1.5 µg mL^−1^) of nine sea cucumber extracts in total (genera *Holothuria*, *Bohadschia* and *Actinopyga*) for (**A**) suspended cells in the water and (**B**) attached to the surface of the incubation flask. Significant differences compared to the control (CT = control) are shown in Table S1.

Measurements of *Chl a* concentration of the suspended cells in the water and the attached cells at the flasks surface showed that the sea cucumbers crude extracts had a concentration-dependent effect on growth and settlement of *C. closterium* (Figure S1A–F). The LRR supports this finding (Figure 3A,B), showing the highest negative effect (*p* < 0.05) on diatom growth in the water column at the highest extract concentrations (150 µg mL^−1^, Figure 3A), except for extracts from *H. whitmaei* and *H. hilla* where no negative effects could be observed (*p = 0.371* and *p = 0.65*, respectively; Table S1). *Actinopyga spp.* and *Bohadschia* extracts (except *B. vitiensis*) exhibited negative LRR at 15 µg mL^−1^ concentration, indicating significant anti-fouling effects. Extracts of the genera *Holothuria* (except *H. atra*) had no inhibitory effects at the same concentration. At the lowest concentration (1.5 µg mL^−1^; Figure 3A), all the crude extracts showed a positive LRR, except *B. argus* and *A. echinites* extracts, which had significant inhibitory activity toward the tested diatom in the water column.

Similar to the LRR in the water column, the highest crude extract concentrations (150 µg mL^−1^) inhibited diatom settlement (Figure 3B). The treatment containing 15 µg mL^−1^ of extract of the genus *Holothuria* stimulated diatom settlement, whereas *Bohadschia* (except *B. vitiensis*) and *Actinopyga* extracts suppressed it. At the lowest concentration (1.5 µg mL^−1^) all crude extracts (except *B. vittiensis*) showed a significant inhibition on diatom settlement (Table S1).

### 2.2. Saponin Profile of the Crude Extracts

#### 2.2.1. Saponin Composition

Identification of the most prominent saponins in the crude extracts of the nine sea cucumber species (peak areas > 10,000 mu) yielded 102 different saponin-like molecules (Table S2). However, several of the saponins showed the same exact molecular mass, but different retention times, indicating unknown isomers of potentially known saponin compounds (Table S3).

**Figure 4:**
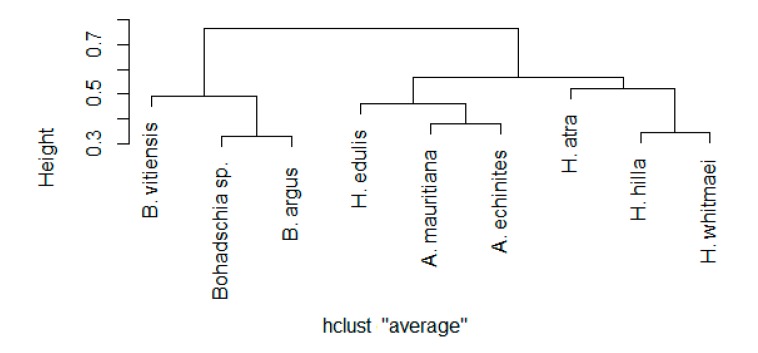
Cluster dendrogram of sea cucumber species based on their studied saponin and sapogenin compositions (“average” distance type, log-transformed data, R version 1.2.5019).

A hierarchical cluster analysis was performed to explore the similarity of saponin compositions between the different holothurian species. Except for *H. edulis*, we observed that all sea cucumber species cluster with species from the same genus (using the Kelley-Gardner-Sutcliffe (KGS) penalty function for identifying significant clusters, Figure 4). Note, that all species from the genus *Bohadschia* formed a clear separated cluster compared to *Actinopyga* and *Holothuria*. 

**Figure 5:**
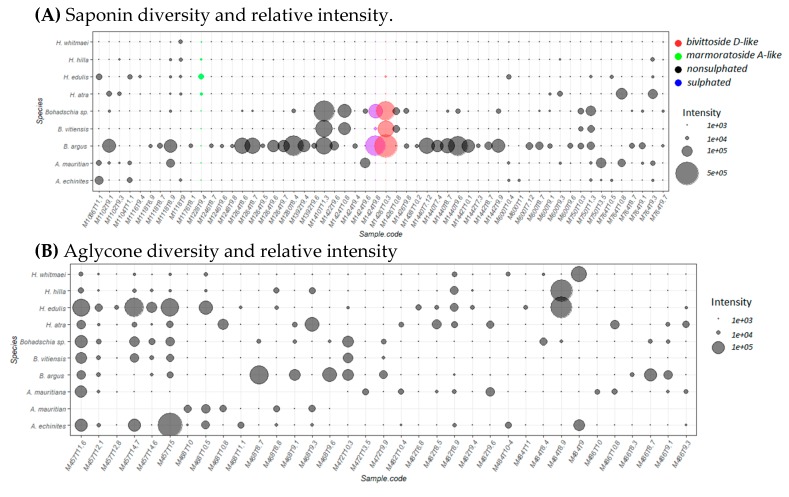
Major saponin compounds detected in the studied sea cucumbers (peak area ≥ 10^4^). (**A**) saponin diversity and relative intensity and (**B**) sapogenin (aglycon) diversity and relative intensity. Sample codes represent exact mass (M in Da), and retention time (T in min). Different colors represent the presence of sulphate groups (in blue), non-sulphate groups (in black) and pure compounds (in purple and red). Bubble size correlates with differences in relative peak areas of the respective molecules.

More detailed analysis of the various saponin compounds revealed that compound M1104T11.1 (abbreviation indicates molecular mass (M) and retention time (T)) was present in all nine sea cucumber species, M1118T8.9 in eight and M600T9.3 and M1374T9 in seven species (*cf.* Table S3). Composition and relative intensities of both saponins and sapogenins, which are visualized for each sea cucumber species (Figure 5A,B), showed that *Bohadschia* species contained the highest number of known saponins, as well as the highest intensities, whereas signal intensities of sapogenins were especially high in the genus *Holothuria*. Interestingly, the three investigated *Bohadschia* species, which were among the most active in inhibiting *C. closterium* growth (Figure 5A), were the only ones containing M1426T10.3 (*m/z* 1426.698; C_67_H_110_O_32_), M1410T11.3 (m/z 1410.703; C_67_H_110_O_31_), and M1424T9.8 (*m/z* 1424.6823; C_67_H_108_O_32_), which represent analogous molecular formulas to the known saponins *bivittoside D-like, bivittoside C-like,* and *marmoratoside A-like*, respectively (Figures S2–S4). 

#### 2.2.2. Total Saponin Concentration

**Figure 6:**
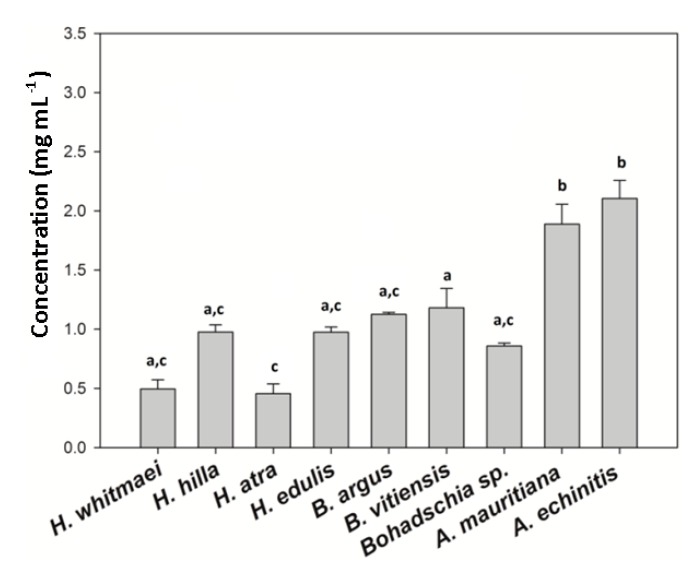
Absolute saponin concentration of the tested crude extracts. (**a**–**c**) indicate significant differences between different sea cucumber crude extracts. Kruskal–Wallis, Dunn’s method as a multiple comparison test. Significance level at *p* < 0.05 was applied.

The total triterpene glycoside concentration of the crude extracts was assessed using the vanillin-sulfuric acid colorimetric assay (Figure 6). *H. atra* (0.456 mg mL^−1^ ± 0.08) and *H. whitmaei* (0.496 mg mL^−1^ ± 0.08) had the lowest saponin concentration, whereas *A. echinites* (2.106 mg mL^−1^ ± 0. 16), *A. mauritiana* (1.880 mg mL^−1^ ± 0.15), *B. vitiensis* (1.181 mg mL^−1^ ± 0.01), and *B. argus* (1.130 mg mL^−1^ ± 0.01) contained the highest concentrations of saponins. Saponin concentration in the genus *Actinopyga* was significantly higher than within *Holothuria* and *Bohadschia* (Kruskal-Wallis test; *p* < 0.05). 

### 2.3. Anti-Fouling Effects of Purified Saponin Fractions and Pure Compounds

#### 2.3.1. AF Assay with an Emphasis on Saponins

**Figure 7:**
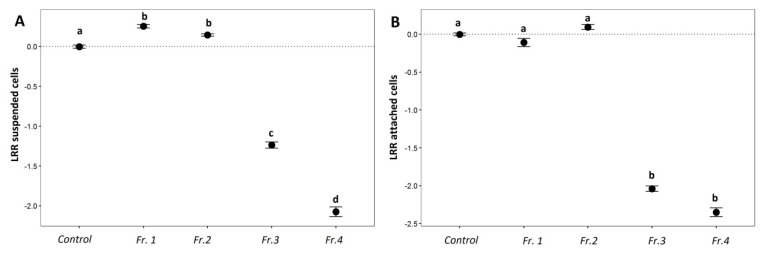
Logarithmic response ratio (LRR) of *C. closterium* following exposure to *B. argus* extract fractions in suspended cells in the water (**A**) and attached to the substrate (**B**). Fr.1 and Fr.2: impure, Fr. 3: semi-pure, Fr. 4: pure singular saponin species (*bivittoside D-like*). a–d represent result of Kruskal-Wallis test; *p* < 0.05.

Based on the LRR of *Chl a* calculated for 1.5 µg mL^−1^ of different fractions, the Kruskal-Wallis test revealed that fraction 3 and 4 had a significant negative effect on the growth of *C. closterium* (*p* < 0.05). The first two fractions, on the other hand, had a significant positive effect on the growth of the diatom species (Figure 7A,B).

#### 2.3.2. Saponin Profile of the Fractions

The most abundant saponin compounds in *B. argus* (i.e., C_67_H_110_O_32_, *bivittoside D-like* and C_67_H_108_O_32_, *marmoratoside A-like*; Figure S5, Table S4) were isolated to examine whether saponins are responsible for the observed anti-fouling activities. As shown in Figure S6, the mixed fraction 3, containing the saponin species M1426T10.3 (*bivittoside D-like*), M1454T10.7 (*stichoposide D-like*) and 1424T10.4 (*marmoratoside A-like*), as well as the relatively pure fraction 4 with mainly saponin M1426T10.3 (*bivittoside D-like*) strongly inhibited growth as well as attachment of the diatom. Fraction 1 and 2, on the other hand, contained a mixture of saponins (except *bivittoside D-like*), which did not affect the growth and attachment of *C. closterium*. Currently, the main three saponin species from fraction 3 are further purified and their molecular structure is being elucidated via NMR spectroscopy. 

## 3. Discussion

Many marine benthic organisms (e.g., sponges, mussels, starfishes, sea urchins, algae) are known to harbor anti-fouling metabolites that protect them from deleterious fouling organisms (e.g., [33,64,65,66,67]). Sea cucumbers do not have visible defensive mechanisms, however their surfaces are free of fouling organisms [40]. Several molecules with various biological activities (e.g., anti-bacterial, anti-fungal, ichthyotoxic) are reported from sea cucumbers, including their anti-fouling properties [38,68]. The AF potential was found to be species specific, and saponins were identified as the main bioactive molecules responsible for these activities [69].

This study demonstrated that the AF properties of the crude extracts of nine sea cucumber species were related to the presence of particular chemical compounds. Our results showed a clear dose-effect for the genus *Actinopyga* and *Bohadschia*, with minimal growth and settlement inhibition at the lowest concentration. Only two of the four tested *Holothuria* species (*H. atra* and *H. edulis*) inhibited algal growth and settlement at the highest concentration, whereas their lower doses (15 and 1.5 µg mL^−1^) actually induced diatom growth, which is following the hormetic effects described by Stebbing ([70,71]; Figure 3). Similar patterns have been reported for crude extracts of *Holothuria leucospilota* against the diatoms *Nitzschia closterium* and *Navicola subinflata*, where lower concentrations (i.e., < 400 µg mL^−1^) of *H. leucospilota* crude extract induced diatom settlement, and at higher concentrations (i.e., > 400 µg mL^−1^) inhibited their growth [69].

Previous studies have shown that steroidal and triterpene glycosides in sponges, gorgonians, sea stars, sea urchins, and sea cucumbers are responsible for the observed anti-fouling activities [15,36,38,41,65,72,73]. Saponins have often been described from holothurians including their various biological activities [74]. For example, studies on *Holothuria glaberrima* [40], *H. atra* and *Holothuria nobilis* [38] showed that saponins were responsible for the observed anti-fouling activities. Also, Selvin and Lipton (2004), and Ozupeck and Cavas (2017) found that the saponin-enriched fraction of different sea cucumbers (i.e., *Holothuria scabra*, *Holothuria polii* and *Holothuria tubulosa*) had pronounced anti-fouling properties [41,75]. In this study, we demonstrated that the composition of saponins is more similar within species of the same genus. For example, the saponin compositions of the genus *Bohadschia* was rather different from the genus *Holothuria* and *Actinopyga* (Figure 4). These observations were in line with the strong AF effects of *B. argus* and *Bohadschia sp.* crude extracts (Figure 3). As apparent from our AF assays, not only *Bohadschia*, but also the genus *Actinopyga* showed much stronger activities compared to *H. atra* and *H. edulis* (Figure 3), which may be explained by significantly higher concentrations of total saponins (*cf.* Figure 6). 

Looking at the saponin profile of the studied genera, we observed similar patterns as described by Kalinin and his colleagues (2015), that non-sulphated saponins with molecular weights of *m/z* 1426.698, and *m/z* 1410.703 were found in the highest intensity in the genus *Bohadschia* [76,77,78]. All these saponins contain six monosaccharide units in their glycone parts, and were present nearly 1–5-fold higher than the tetraosides (*m/z* 1118.551 and *m/z* 1102.556 ), which were present in the other sea cucumber species. Whereas, sulphated saponins (e.g., molecular weights of *m/z* 1206.510 and *m/z* 868.389), putatively annotated as *echinoside A* and *echinoside B* respectively, were observed only in the two genera of *Actinopyga* and *Holothuria*. This is in accordance with the results from Kitagawa and colleagues (1981; 1989), and Grauso and colleagues (2019), who reported *echinoside A* and *B* from *Holothuria* (i.e., *H. atra;* [79]), and *Actinopyga* species [80]. *Actinopyga* and *Holothuria* extracts also contained mixtures of biosides including *bivittoside A* like compounds (C_41_H_66_O_12_; *m/z* 750.455), tetraosides such as the saponin *desholothurin A* (C_54_H_86_O_24_; *m/z* 1118.551) and *pervicoside B* (C_54_H_86_O_22_; *m/z* 1086.561). Our data indicated that the AF activity may correlate with the amount and/or type of sugar units in their glycone part (in genus *Bohadschia*). A similar result has been observed by Van Dyck and colleagues (2010), who analyzed the saponin profile of *Holothuria forskali* in undisturbed and under predator stress conditions [43]. In the undisturbed state, the body wall of *H. forskali* produced mainly tetraosides (i.e., *holothurinoside C* (*m/z* 1102) and *desholothurin A* (*m/z* 1118)), while under stressed conditions *holothurinoside C* was converted to the hexaosides *holothurinoside F* (*m/z* 1410) and *holothurinoside H* (*m/z* 1440) and *desholothurin A* was converted to the hexaosides *holothurinoside* G (*m/z* 1426). However, Van Dyck and colleagues (2010) also pointed out that *m/z* 1426 is produced under both environmental states in the tested sea cucumber, suggesting that *m/z* 1426 is a “background prevention signal,” and other molecules might play more important roles under stressful conditions [81]. Also, Kalinin and colleagues (2015) described a molecule with the same molecular mass, but different side chain (*m/z* 1426) as a characteristic saponin of *Bohadschia*, which was identified as “*bivittoside D.*” A remarkable similarity observed between *H. forskali* and genus *Bohadschia* is the presence of chemically defended CTs (*cf*. Figure 1), each containing different saponin mixtures [43,82].

The mechanism of action of many extracted and isolated molecules with anti-fouling effects are usually unclear because of multiple possible interactions involved [83]. As mentioned earlier, saponins are amphiphilic molecules with hydrophilic and hydrophobic properties. The amount of monosaccharides attached on the C-3 position (Figure 2) of the steroid affects the hydrophilicity of the saponin molecule, which can affect the permeability of the cell membranes by inducing curvature and forming pores in the membrane [84]. Therefore, high integration values of hexaosidic saponins, containing lanosterol as the major sterol within the genus *Bohadschia* [85], may explain their strong AF activities [81].

It can be concluded that the AF activity is species-specific in sea cucumbers and related to not only total saponin concentration (e.g., in *Actinopyga*), but also saponin composition (such as shown in *B. argus*). AF activities of the studied crude extracts showed that *B. argus* contained compounds affecting fouling by the diatom *C. closterium*. Consequently, purified fractions and pure compounds of *B. argus* (Figure 7) confirmed that particular saponin compounds (here *m/z* 1426.698) had strong inhibitory effects on growth and settlement of the diatom *C. closterium*. Furthermore, the here performed anti-fouling assays can be a promising and fast method for identifying compounds with anti-fouling activity and for pre-selecting bioactive extracts and/or compound from various organism to discover ecologically and potentially pharmaceutically active natural products.

## 4. Materials and Methods

In this study we investigated nine holothurian species from the family Holothuriidae that were collected from Guam in 2016. These nine species were members of three different genera, *Holothuria (H. whitmaei, H. hilla, H. atra, H. edulis)*, *Bohadschia (B. argus, B. vittiensis, Bohadschia sp.)* and *Actinopyga (A. echinites, A. mauritiana;* Figure 2). 

### 4.1. Experimental Setup

#### 4.1.1. Cylindrotheca Closterium Culture

The AF assays were conducted using the diatom species *C. closterium* as the test organism. The diatom cultures were kept in climate chamber with constant temperature of 18 °C with a light and dark cycle of 12 h and a light intensity of 90 µmol m^−2^ s^−1^. The initial stock culture (strain number CCAP-1017/8) was obtained from Culture Collection of Algae and Protozoa (CCAP), and was prepared in 250 mL polystyrene culture flasks, filled with sterile artificial seawater enriched with F⁄2 nutrients, which has shown to be an optimal nutrient supply for this algal species [86,87].

#### 4.1.2. Preparation of Sea Cucumber Crude Extracts

Extractions of sea cucumbers were performed by freeze dried material. For each extraction a 1:10 ratio (w/v) of freeze-dried sea cucumber tissue (in g) and organic solvent mixture (in mL) was used. In brief, the ground tissue samples were extracted twice with a 1:1 mixture (v/v) of methanol (MeOH) and ethyl acetate (EtOAc) and a third and final time with 100% MeOH. Samples were shaken for at least 3 h during each subsequent extraction. After filtering through filter paper (Diameter: 150 mm, Grade: 3 hw, Sartorius GmbH, 37979, Goettingen, Germany), extracts were dried by rotary evaporation (Rotavapor RII, BUCHI, Flawil, Switzerland) and finally transferred and dried using a centrifugal vacuum concentrator (Speedvac, Christ RVC 2–25 Co plus; Freeze dryer: Christ Alpha 2–4 LD plus). The dried crude extracts were weighted and stored at −20 °C until further usage. 

#### 4.1.3. Anti-Fouling Assay: Experimental Design

**Figure 8:**
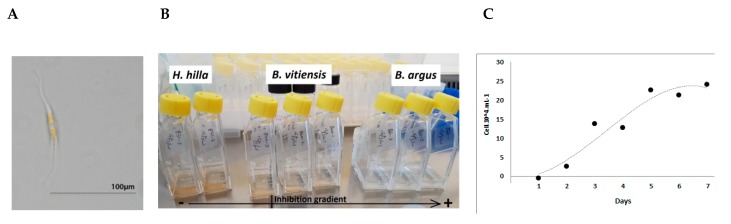
(**A**–**C**). The test organism *C. closterium* under the microscope (**A**), culture flasks demonstrating high growth rates (left, not-inhibited), medium growth rates (middle) and low growth rates (inhibited) of *C. closterium* (**B**), growth curve of *C. closterium* in 7 days in the stock solution (**C**).

The effect of the sea cucumber crude extracts on the growth and settlement behavior of *C. closterium* was tested by monitoring the biomass of the diatom after 24 and 72 h incubation, based on chlorophyll a (*Chl a*) concentration of suspended cells in the water as well as attached cells on the flask surface. The AF assays were performed in 40 mL culture flasks (TC Flask T25, SARSTEDT AG & Co. KG, 51588, Nümbrecht, Germany). All crude extracts were dissolved in MeOH and added in triplicates to empty the cell culture flasks in order to obtain three different final concentrations of the crude extracts (150, 15 and 1.5 µg mL^−1^, Figure 8). After the MeOH evaporated, 10% of the diatom stock (i.e., 1.5 mL of algae inoculated in 15 mL F/2 medium; OD_442_ = 0.46 ± 0.01) was inoculated to the culture flask pre-filled with 18 mL of sterile artificial seawater. For three days, the flasks were stored horizontally in a growth chamber under the above-mentioned culturing conditions (Section 4.1.1) to perform the diatom surface attachment experiment. Treatments with only MeOH and no sea cucumber crude extract served as control experiment.

The potential AF effects of particular saponin species were assessed using fractions isolated from *B. argus*. The assay with the purified saponin fraction and pure saponin compounds were conducted with only the lowest concentrations of 1.5 µg mL^−1^. 

#### 4.1.4. Diatom Growth and Settlement Analyses

*Chlorophyll a measurements:* To assess diatom biomass, *Chl a* was extracted from the water samples after 24 and 72 h of inoculation. Furthermore, to study the attachment behavior of the diatom, the *Chl a* content of *C. closterium* attached to the substrate was extracted after 72 h (end of the experiment), except for the highest concentration. Since we observed that algae biomass was dramatically reduced in most of the extracts exposed to the highest extract concentration (150 µg mL^−1^), *Chl a* concentrations in both, suspended in water and attached to substrate, were measured only after 24 h of inoculation. Experimental procedure included filtering water samples through a combusted and acid-washed glass microfiber filter (GF/C, Whatman, GE Healthcare life sciences, Pittsburg, PA 15264-3065, USA) and storing at −80 °C until extraction. For extraction, ethanol (90%) was added to the samples, vortexed, and then placed in an ultrasonic bath filled with ice for 30 min. Before measuring pigment concentrations, all samples were stored for 24 h at 4 °C. Measurements were conducted with a microplate reader (BioTek, SYNERGY H1, Winooski, VT, USA) to determine the *Chl a* concentration using a fluorescence excitation (Ex) wavelength of 395 nm and emission (Em) wavelength of 680 nm. *Chl a* concentrations were obtained by converting fluorescence data to concentrations using a *Chl a* standard from *Anacystis nidulans* algae (Product Number C 6144, Sigma-Aldrich, St. Louis, MO, USA).

#### 4.1.5. Anti-Fouling Effects: Data and Statistical Analyses

Statistical analyses were performed with R (version 1.1.423, R Foundation for Statistical Computing, Vienna, Austria), and SPSS (Version 26, IBM, NY 10504, USA). We assessed the effect of different sea cucumber extracts and concentrations on diatom settlement, as well as cell density of the diatom *C. closterium.* After testing for normality and homoscedastity, Kruskal-Wallis test was conducted for each extract concentration, followed by Kruskal–Wallis post hoc test. The same method was applied for the purified fractions and pure compounds (Section 4.3). Differences were considered significant at a 95% confidence level. The logarithmic response ration (LRR; Equation (1)) was calculated as the ratio of *Chl a* concentration affected by crude extracts to the controls. LRR > 0 illustrates higher *Chl a* concentration and thus a positive effect in extract treatments, while LRR < 0 identifies decreased *Chl a* concentrations, and thus a negative effect compared to control samples.

(1) $$\mathrm{LRR}=Ln\left(\frac{treatment}{control}\right)$$

### 4.2. Saponins as Potential Bioactive Compounds Affecting the Fouling Organism C. closterium

#### 4.2.1. Dereplication of Saponins

To analyze the content of the most abundant saponin species within the different sea cucumber crude extracts (dissolved in MeOH), an aliquot was analyzed using ultra performance liquid chromatography-high resolution mass spectrometry (UPLC-HRMS; Tables S2 and S3). Chromatographic separation was achieved on a Waters Acquity BEH C_18_ column (1.7 µm, 2.1 mm × 50 mm) with an ACQUITY ultra performance liquid chromatography (UPLC) H-Class System (Waters Co., Milford, MA, USA) coupled to a Synapt G2-Si HDMS high-resolution Q-ToF-MS (Waters Co., Manchester, UK) equipped with a LockSpray dual electrospray ion source operated in positive (POS) ionization modes. The Q-ToF-MS was calibrated in resolution mode over a mass-to-charge (*m/z*) ranging from 50 to 2000 Dalton by using a 0.5 mmol L^−1^ sodium formate solution. For each run leucine enkephalin was used as the lock mass, generating a reference ion for POS mode ([*m/z* 556.277 M + H]^+^) to ensure a mass tolerance for all LC-MS or LC-MS/MS experiments of less than one ppm. Mass spectral data were collected using the MS^e^ data acquisition function to simultaneously obtain information on the intact molecule (no collision energy applied) as well as their fragmentation data (collision energy ramp reaching from 15 to 75 eV). Analytes were eluted at a flow rate of 0.6 mL min^−1^ using a linear gradient of milliQ water (H_2_O, 100%, eluent A) to acetonitrile (ACN, 100%, eluent B) both with 0.1% formic acid. The initial condition was 100% A held for 0.5 min, followed by a linear gradient to 100% B in 19 min. The column was then washed with 100% B for 9.5 min and subsequently returned and held for 2.9 min to the initial conditions (100% eluent A) to equilibrate the column for the following run. The column temperature was set to 40 °C.

*Data treatment:* To identify different saponin compounds in the holothurian extracts we compared the molecular masses of known saponins to the here-analyzed mass data (MS^1^) and by confirmation the saponin nature (Figure 1) by identifying their diagnostic key fragments. Therefore, we used different diagnostic key fragments corresponding to oligosaccharides residues [88], and the sapogenin molecule (aglycone) part (Table 1). Unknown saponin molecules (with different molecular formulas than previously reported) were not considered in this analysis. Given that we identified several saponins with the same exact mass (probably isomers), we retained the following information for compound identification: (1) retention time (RT), (2) molecular weight and (3) the integrated area of the respective peak (Table S3). 

#### 4.2.2. Saponin Compounds Composition: Data and Statistical Analyses

The integrated areas have been log transformed to reduce the skewness. Principal component analysis (PCA) was used to evaluate the differences between saponin compositions of the studied sea cucumbers. In order to identify the saponin similarity among different sea cucumber species, a hierarchical cluster analysis (function *hclust*, using packages ape for R) was used. After choosing the best cluster method using cophenetic correlation distances (pearson correlation), the penalty function of Kelley Gardner Sutcliffe (*KGS*; package maptree in R) was used to trim the dendrogram. Compounds with integration values higher than 10,000 were then selected to further study the saponin composition of each of the sea cucumber species.

#### 4.2.3. Total Saponin Concentration within the Examined Sea Cucumber Species

Since only known saponins could be identified by the LC-MS/MS data, we also quantified total saponin concentration of different sea cucumbers using a spectrophotometric method with vanillin-sulfuric acid, which was adapted after Hiai and colleagues [98]. Based on their method, sulfuric acid oxidizes saponins and transformes glycone chains to furfural. The free hydroxyl group at the C-3 position of the agylcone part reacts with vanillin and produces a distinctive yellow-brown color [41]. According to this methodology, we prepared 8% vanillin solution (w/v) dissolved in ethanol (analytical grade), and sulfuric acid 72% (v/v) dissolved in distilled water. Crude extracts as well as double distilled water (used as blanks), were mixed with vanillin (8%; AppliChem GmbH, Germany) and sulfuric acid (72%) in a 1:1:10 (v/v/v) proportion in an ice bath. Next, we incubated the obtained solution at 60 °C in a water bath for 10 min. To stop the reaction, samples were cooled down on ice. A standard curve was measured, using a concentration gradient of Quillaja bark saponin (AppliChem GmbH, 64291, Darmstadt, Germany), diluted in distilled water. Finally, the absorbance was measured at 540 nm using a microplate reader. 

### 4.3. Anti-Fouling Effects of Purified Saponin Fractions

We further fractionated the crude extract of *B. argus*, since it had exhibited one of the highest AF activity among the tested organic extracts. The aim was the identification of one or multiple saponin compounds responsible for the anti-fouling activity observed in the crude extract. 

### 4.4. Sample Fractionation and Purification

*Liquid/liquid partitioning:* The crude extracts of *B. argus* were first partitioned using (1) EtOAc:H_2_O (1:1) followed by partitioning of the H_2_O fraction with (2) n-BuOH:H_2_O (1:1). 

**Figure 9:**
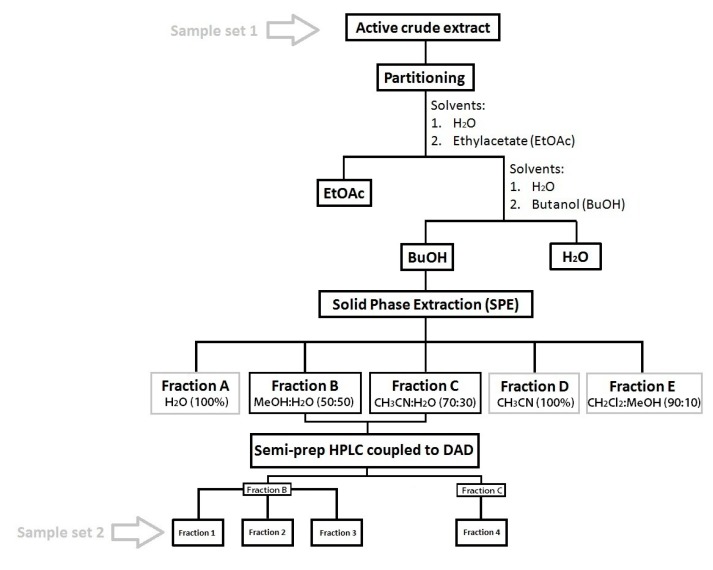
Flow chart showing the applied procedure for isolating the bioactive saponin compounds (Cutignano et al., 2015; Ebada et al., 2008 [99,100]). Sample set 1 and 2 refers to the samples that were tested for anti-fouling (AF) activity in this study.

*Solid Phase Extraction (SPE) chromatography:* The BuOH fraction which contained the saponins was further fractionated by SPE chromatography [99]. Therefore, the SPE column (SUPELCLEAN LC_18_, 60 mL/10 g; Supleco Park, USA) was desalted/washed with 60 mL MeOH and preconditioned with 120 mL distilled water. Then, the concentrated BuOH fraction was added to the column and washed with five elution gradients: (1) Elution with H_2_O (Fraction A, 120 mL), (2) MeOH:H_2_O (Fraction B, 50:50, 180 mL), (3) ACN: H_2_O (Fraction C, 70:30, 180 mL), (4) ACN 100% (Fraction D, 180 mL) and (5) CH_2_Cl_2_: MeOH (fraction E, 90:10, 180 mL; Figure 9).

*Preparative HPLC:* Preliminary biological and chemical screening of each SPE fraction showed that fractions B (MeOH:H_2_O 50:50) and C (CH_3_CN:H_2_O 70:30) contained not only diverse and high amounts of saponins, they also had high activities against the fungi *Rhodotorula glutinis* and *Candida albicans* (unpublished data). Therefore, these fractions were selected for further purification by semi-preparative HPLC (Agilent Technologies, 1260 Infinity) with a PDA detector (Agilent, G4212-60008, CA, USA). Chromatographic separation was achieved using a C_18_ column (Pursuit XRs 5 µm, 250 mm × 10 mm, Agilent, CA, USA) with a pre-column (2.7 µm, 2.1 mm × 5 mm, Agilent, CA, USA) and applying a linear gradient: initial 50% A/50% B, 0–4 min 50% A/50% B; 4–36 min 38% A/62% B; 36–39 min 100% B, and a column reconditioning phase for 39–59 min 100% B, and 8 min to 50% A/50% B. (flow rate 1.5 mL min^−1^; eluent **A:** 95% H_2_O and 0.1% of formic acid 98% (Roth); eluent **B:** ACN and 0.1% formic acid). Several fractions were collected by peak picking at specific retention times. In order to determine the saponin composition of the obtained fractions and pure compounds, the fractions and compounds were dissolved in HPLC-grade MeOH, filtered through a 0.2 µm syringe filter, and injected into the HPLC-DAD-MS system, as previously described in Section 4.2.1. The peak integration of saponins in the final fractions has been assessed (Table S4), and these fractions have been used for AF assay. 

## Figures and Tables

**Table 1:** Key diagnostic fragments of saponins detected via the MS/MS analysis of the studied sea cucumbers.

Diagnostic Ions	Reported Exact Mass *(m/z)*	Molecular Formula	Organism	References
Sapogenin	472.3552	C_30_H_48_O_4_	*B. vitiensis*	[89]
Sapogenin 1	482.3032	C_30_H_42_O_5_	*Octacoral (Anthomastus bathyproctus)*	[90]
Sapogenin 3	457.3318	C_29_H_45_O_4_	*Gorgonian (Eunicella cavolini)*	[91]
Caudinoside A	468.3239	C_30_H_44_O_4_	*Paracaudina ransonetii*	[92]
Stichopogenin A4	486.3345	C_30_H_46_O_5_	*Stichopus japonicus*	[93]
16 Keto holothurinogenin	484.3189	C_30_H_44_O_5_	*A. mauritiana*	[94,95]
MeGlc-Glc-Qui + Na^+^	507.164	C_19_H_32_O_14_Na^+^	*H. lesson, H. forskali*	[96,97]

## Supplementary Materials

Figure S1A–F: *Chl a* concentrations in the suspended cells in the water after incubation of *C. closterium* with different concentrations of sea cucumbers extracts (A = 150 µg mL^−1^; C = 15 µg mL^−1^; E = 1.5 µg mL^−1^) and of *C. closterium* attached to the flask surface (B = 150 µg mL^−1^; D = 15 µg mL^−1^; F = 1.5 µg mL^−1^). Dashed lines separate different genera of sea cucumbers (*Holothuria, Bohadschia, Actinopyga*). CT = Control. (a–e) indicate significance levels according to post hoc test. Figure S2: LC/MS spectra of the crude extracts of genus *Holothuria* (Y-axis relative intensity in % of maximum peak, x-axis retention time in minutes). Figure S3: LC/MS spectra of the crude extracts of genus *Bohadschia* (Y-axis relative intensity in % of maximum peak, x-axis retention time in minutes). Figure S4: LC/MS spectra of the crude extracts of genus *Actinopyga* (Y-axis relative intensity in % of maximum peak, x-axis retention time in minutes). Figure S5: LC/MS spectra of fractions isolated from *B. argus* (see Table S4; Y-axis relative intensity in % of maximum peak, x-axis retention time in minutes). Figure S6: Identified saponins species presented in different fractions isolated from *B. argus.* The red color referred to the presence of a semi-purified saponin species (*bivittoside D-like* at *m/z* 1426.698). Size of bubbles represented the peak area of the molecules obtained from LC/MS analysis. Table S1. Significant differences (reported as *p-values*) of sea cucumber crude extracts compared to control experiments using the Kruskal–Wallis test. Table S2: Saponins reported, and found in studied species. Table S3: Exact mass (*m/z*), molecular formula, retention time (RT), and intensity signal (IntSig) of saponins, and sapogenins (aglycone parts) presented in the three sea cucumber genera *Holothuria*, *Bohadschia* and *Actinopyga*. Table S4: Exact mass (*m/z*), molecular formula, retention time (RT in minutes) and intensity signal of saponins presented in isolated fractions of *B. argus.*
