# Supplementary material for: Anti-Fouling Effects of Saponin-Containing Crude Extracts from Tropical Indo-Pacific Sea Cucumbers
Source: Mar Drugs. 2020 Mar 31;18(4):181. doi: 10.3390/md18040181 (PMC7231054; doi:10.3390/md18040181)
Supplement: Supplementary file 1 [file marinedrugs-18-00181-s001.pdf]

Table S1. Significant differences (reported as *p-values*) of sea cucumber crude extracts compare to control experiments using the Kruskal-Wallis test.

Conc. (µg mL^{-1})	Species / Condition	*A. mauritiana*	*A. echinites*	*Bohadschia sp.*	*B. vitiensis*	*B. argus*	*H. edulis*	*H. atra*	*H. hilla*	*H. whitmaei*
**150**	**suspended**	0.000	0.000	0.000	0.000	0.000	0.000	0.050	0.371	0.649
	**attached**	0.011	0.000	0.000	0.000	0.000	0.000	0.000	0.091	0.398
**15**	**suspended**	0.000	0.001	0.010	0.794	0.006	0.374	0.011	0.709	0.052
	**attached**	0.002	0.036	0.098	0.050	0.121	0.385	0.003	0.000	0.008
**1.5**	**suspended**	0.179	0.001	0.174	0.159	0.025	0.883	0.128	0.000	0.001
	**attached**	0.000	0.000	0.000	0.859	0.001	0.000	0.017	0.000	0.011

Table S2. Saponins reported, and found in studied species

**Exact mass (*m/z*)**	**Molecular formula**	**Compound**	**Species**	**Bioactivity**	**Reference**
600.3662	$C_{35}H_{52}O_{8}$	*hillaside A*	*Holothuria hilla*	Cytotoxic	(Wu et al. 2007)
694.3928	$C_{37}H_{58}O_{12}$	*hillaside B*	*H. hilla*	Cytotoxic	Wu et al., 2007
750.4554	$C_{41}H_{66}O_{12}$	*bivittoside A*	*Bohadschia bivittata*[*1]	Anti-fungal	(Kitagawa et al. 1989a)
764.4347	$C_{41}H_{64}O_{13}$	*holothurinosideD*	*H. Forskali*	Anti-viral	(Rodriguez et al. 1991)
806.4453	$C_{43}H_{66}O_{14}$	*stichorrenoside C*	*Stichopus horrens*	n.d.	(Cuong et al. 2017)
868.3891	$C_{41}H_{65}NaO_{16}S$	*echinoside B*	*Achtinopyga echinites, Actinopyga mauritiana*	Anti-fungal	(Kitagawa et al. 1980)
870.3683	$C_{40}H_{63}NaO_{17}S$	*hillaside C*	*H. hilla*	Cytotoxic	(Wu et al. 2006b)
882.3683	$C_{41}H_{63}NaO_{17}S$	*holothurin B*	*Holothuria leucospilota*	Ichthyotoxin	(Kitagawa et al. 1978a)
		holothurin B4	*Holothuria polii*	n.d.	(Silchenko et al. 2005a)
		nobiliside B	*Holothuria nobilis*	cytotoxic	(Wu et al. 2006a)
884.384	$C_{41}H_{65}NaO_{17}S$	*holothurin B2*	*H. polii*	Taxonomic marker	(Silchenko et al. 2005b)
		leucospilotaside B	*H. leucospilota*	Cytotoxic	(Han et al. 2010)
984.3071	$C_{41}H_{62}Na_{2}O_{20}S_{2}$	*lecanoroside A*	*Actinopyga lecanora*	Cytotoxic	(Zhang et al. 2008)
1054.5348	$C_{53}H_{82}O_{21}$	*cladoloside A*	*Cladolabes sp*	n.d	(Avilov and Stonik 1988)
		cladoloside A4	*Cladolabes schmeltzii*	Cytotoxic	(Silchenko et al. 2014)
1086.561	$C_{54}H_{86}O_{22}$	*DS-pervicoside B*	*Holothuria pervicax*	Anti-fungal	(Kitagawa et al. 1989b)
1088.5767	$C_{54}H_{88}O_{22}$	*bivittoside B*	*B. bivittata*	Anti-fungal	(Kitagawa et al. 1981a; Kitagawa et al. 1989a)
		DS-pervicoside C	*H. pervicax*	Anti-fungal	(Kitagawa et al. 1989b)
1100.5767	$C_{55}H_{88}O_{22}$	*lefevreioside A1*	*Cucumaria lefevrei*	n.d.	(Rodriguez and Riguera 1989)
		cucumarioside A15	*Eupentacta fraudatrix*	Anti-fungal, Cytotoxic	(Silchenko et al. 2012)
1102.5559	$C_{54}H_{86}O_{23}$	*holothurinoside C*	*H. Forskali*	Anti-tumour, anti-viral	(Rodriguez et al. 1991)
1102.5559	$C_{54}H_{86}O_{23}$	*apostichoposide A1*	*Apostichopus japonicus*	Cytotoxic	(Zhang et al. 2020)
1104.5353	$C_{53}H_{84}O_{24}$	*holothurinosideX*	*Holothuria lessoni*	n.d.	(Bahrami et al. 2016)
1104.5716	$C_{54}H_{88}O_{23}$	*holothurinosideY*	*H. lessoni*	n.d.	(Bahrami et al. 2018)
		DS-echinoside A	*Pearsonothuria graeffei*	Anti-cancer	(Zhao et al. 2011)
1116.5353	$C_{54}H_{84}O_{24}$	*apostichoposide C*	*A. japonicus*	n.d.	(Zhang et al. 2018)
1118.5509	$C_{54}H_{86}O_{24}$	*desholothurin A*	*H. Forskali*	Anti-tumour	(Rodriguez et al. 1991)
		arguside E	*Bohadschia argus*	Cytotoxic	(Liu et al. 2008b)
1120.5666	$C_{54}H_{88}O_{24}$	*holothurinosideZ*	*H. lessoni*	n.d.	(Bahrami et al. 2014a)
1144.5665	$C_{56}H_{88}O_{24}$	*arguside A*	*B. argus*	cytotoxic	(Liu et al. 2007)
1166.5178	$C_{54}H_{86}O_{25}S$	*perivicoside B*	n.d.	antibiotic	(Kokai Tokkyo Koho 1984)
1168.5335	$C_{54}H_{88}O_{25}S$	*perivicoside C*			

1176.5563	$C_{56}H_{88}O_{26}$	*arguside D*	*B. argus*	Anti-fungal	(Liu et al. 2008a)
1198.4841	$C_{55}H_{83}NaO_{25}S$	*intercedenside A*	*Mensamaria intercedens*	Anti-tumore; cytotoxic	(Zou et al. 2003)
		cucumarioside G3	*E. fraudatrix*	n.d.	(Kalinin et al. 1992)
1200.4998	$C_{55}H_{85}NaO_{25}S$	*cucumarioside G1*	*Cucumaria fraudatrix*[*2]	n.d.	(Afiyatullov et al. 1985)
		violaceuside A	*Pseudocolochirus violaceus*	Cytotoxic	(Zhang et al. 2006b)
1204.4948	$C_{54}H_{85}NaO_{26}S$	*24- dehydroechinoside A*	*Actinopyga agassizi*	n.d.	(Kitagawa et al. 1982)
		fuscocineroside B/C	*Holothuria fuscoscinera*	Cytotoxic	(Zhang et al. 2006a)
		scabraside A	*H. scabra*		(Han et al. 2009)
1206.5104	$C_{54}H_{87}NaO_{26}S$	*echinoside A*	*A. echinites, A. mauritiana*	Anti-fungal	(Kitagawa et al. 1980)
1206.474	$C_{53}H_{85}NaO_{27}S$	*holothurin E*	*H. lesson*	n.d.	(Bahrami and Franco 2015)
1207.4818	$C_{53}H_{84}NaO_{27}S$	*lecanoroside B*	*Actinopyga lecanora*	Cytotoxic	(Zhang et al. 2008)
1214.4791	$C_{55}H_{83}NaO_{26}S$	*intercedenside H*	*M. intercedens*	Cytotoxic	(Zou et al. 2005)
		colochiroside B3	*Colochirus robustus*	cytotoxic	(Silchenko et al. 2015)
1220.4896	$C_{54}H_{85}NaO_{27}S$	*holothurin A*	*H. leucospilota*	Anti-bacteria	(Kitagawa et al. 1979)
			Holothuria edulis	Anti-fungal	(Kobayashi et al. 1991)
			H. polii, Holothuria tubolosa	n.d.	(Silchenko et al. 2005b)
		17-hydroxy fuscocineroside B	*Bohadschia marmorata*	Anti-fungal	(Yuan et al. 2009b; Yuan et al. 2009c)
		25-hydroxy fuscocineroside B			
		25-hydroxy-dehydroechinoside A	*Actinopyga flammea*	Anti-fungal, Anti-tumore	(Bhatnagar et al. 1985)
		22-hydroxy-24-dehydro-echinoside A			
		scabraside B	*Holothuria scabra*	Cytotoxic	(Han et al. 2009)
1222.5053	$C_{54}H_{87}NaO_{27}S$	*holothurin A1*	*Holothuria floridana,*	n.d.	(Oleinikova et al. 1982)
			Holothuria grisea		
		holothurin A4	*H. scabra*	Cytotoxic	(Hal Dang et al. 2007)
1228.4277	$C_{53}H_{80}O_{28}$S2	*cucumechinoside B*	*Cucumaria echinata*[*3]	Anti tumore; anti fungal	(Miyamoto et al. 1990)
1230.474	$C_{55}H_{83}NaO_{27}S$	*intercedenside C,D*	*M. intercedens*	Anti tumore; cytotoxic	(Zou et al. 2003; Zou et al. 2005)
1232.4896	$C_{55}H_{85}NaO_{27}S$	*intercedenside F*	*M. intercedens*	cytotoxic	(Zou et al. 2005)
1234.4689	$C_{54}H_{83}NaO_{28}S$	*calcigeroside C1*	*Pentamera calcigera*	n.d.	(Avilov et al. 2000)
1236.4846	$C_{54}H_{85}NaO_{28}S$	*holothurin D*	*H. lesson*	n.d.	(Bahrami et al. 2014b)
1246.5982	$C_{60}H_{94}O_{27}$	*coustesides G*	*Bohadschia cousteaui*	Anti-fungal	(Elbandy et al. 2014)
1248.6138	$C_{60}H_{96}O_{27}$	*impatienside B*	*H. axiloga*	Anti-fungal	(Yuan et al. 2009a)
1248.6139	$C_{60}H_{96}O_{27}$	*coustesides H*	*B. cousteaui*	Anti-fungal	(Elbandy et al. 2014)
1250.6295	$C_{60}H_{98}O_{27}$	*coustesides J*			
1260.428	$C_{53}H_{82}$Na2$O_{27}S_2$	*magnumoside C_4*	*Massinium magnum*[*4]	Anti-tumour	(Silchenko et al. 2017)
1262.5002	$C_{56}H_{87}NaO_{28}S$	*fuscocineroside A*	*H. fuscoscinerea*	Cytotoxic	(Zhang et al. 2006a)
1264.6088	$C_{60}H_{96}O_{28}$	*causteside E,F*	*B. cousteaui*	Anti-fungal	(Elbandy et al. 2014)
		griseaside A	*H. grisea*	Anti-tumor	(Sun et al. 2008)
		holothurinoside E	*H. forskali*	n.d.	(van Dyck et al. 2010)
1264.5158	$C_{56}H_{89}NaO_{28}S$	*22-acetoxy echinoside A*	*A. flammea*	n.d.	(Bhatnagar et al. 1985)
1266.6245	$C_{60}H_{98}O_{28}$	*coustesides I*	*B. cousteaui*	Anti-fungal	(Elbandy et al. 2014)
		griseaside A	*H. grisea*	Cytotoxic	(Sun et al. 2008)
1280.6037	$C_{60}H_{96}O_{29}$	*holothurinosideA*	*H. Forskali*	Anti-tumour	(Rodriguez et al. 1991)
		causteside C	*B. cousteaui*	Anti-fungal	(Elbandy et al. 2014)
1308.635	$C_{62}H_{100}O_{29}$	*pervicoside D*	*H. axiloga*	Anti-fungal	(Yuan et al. 2009a)
1322.6142	$C_{62}H_{98}O_{30}$	*holothurinoside B*	*H. Forskali*	Anti-viral	(Rodriguez et al., 1991)
		causteside D	*B. cousteaui*	Anti-fungal	(Elbandy et al., 2014)

1374.4856	$C_{59}H_{90}O_{32}S2$	*cucumarioside A3*	*Cucumaria***	Cytotoxic	(Drozdova et al. 1997)
		cucumarioside A6-2			
1386.5138	$C_{60}H_{92}Na2O_{31}S$	*synaptoside A*	*Synaptida***	Cytotoxic	(Avilov et al. 2008)
1392.6561	$C_{66}H_{104}O_{31}$	*holotoxin A1(stichoposide A)*	*Stichopus japonicus*	n.d.	(Elyakov 1983)
1410.703	$C_{67}H_{110}O_{31}$	*bivittoside C*	*B. argus, B. bivittata*	Anti-fungal	(Kitagawa et al. 1981a; Kitagawa et al. 1989a)
1410.703					
1422.6667	$C_{67}H_{106}O_{32}$	*coustesides B*	*B. cousteaui*	Anti-fungal	(Elbandy et al., 2014)
		holotoxin A	*S. japonicus*	Anti-fungal	(Kitagawa et al. 1978b)
1424.682	$C_{67}H_{108}O_{32}$	*marmoratoside A*	*B. marmorata*	Anti-fungal	(Yuan et al., 2009)
		impatienside A	*Holothuria impatiens*	Cytotoxic	(Sun et al. 2007)
1425.3459+	$C_{54}H_{85}NaO_{28}S$	*holothurin A3*	*H. scabra*	Cytotoxic	(Hal Dang et al. 2007)
1426.698	$C_{67}H_{110}O_{32}$	*bivittoside D*	*B. argus, B. bivittata*	Anti-fungal	(Kitagawa et al. 1981a; Kitagawa et al. 1989a)
			Bohadschia vitiensis	Spermicidal, Anti-fungal	(Lakshmi et al. 2008; Lakshmi et al. 2012)
		holothurinoside G	*H. forskali*	n.d.	(van Dyck et al. 2010)
1438.698	$C_{68}H_{110}O_{32}$	*stichoposide C*	*Holothuria***	n.d.	(Stonik et al. 1983)
		stichloroside C1	*Stichopus chloronotus*	Anti-fungal	(Kitagawa et al. 1981b)
1440.677	$C_{67}H_{108}O_{33}$	*marmoratoside B*	*B. marmorata*	Anti-fungal	(Yuan et al. 2009c)
		17-hydroxy impatienside A	*B. marmorata*	Anti-fungal	(Yuan et al. 2009c)
		cousteside A	*B. cousteaui*	Anti-fungal	(Elbandy et al. 2014)
1440.3257	$C_{53}H_{79}Na3O_{35}S_3$	*fallaxoside D_1*	*Cucumaria fallax*	n.d.	(Silchenko et al. 2016)
1442.6929	$C_{67}H_{110}O_{33}$	*arguside B*	*B. argus*	Anti-fungal	(Liu et al. 2008c)
1442.6929	$C_{67}H_{110}O_{33}$	*arguside C*	*B. argus*	Anti-fungal	(Liu et al. 2008c)
1470.6879	$C_{68}H_{110}O_{34}$	*lessonoside A,B,D*	*H. lessoni*	n.d.	(Bahrami and Franco 2015)
1484.7034	$C_{69}H_{112}O_{34}$	*25-acetoxybivittoside D*	*B. marmorata*	Anti-fungal	(Yuan et al. 2009c)

**based on WoRMS: [1] Bohadschia bivittata was not found. Holothuria bivittata is not accepted, and replaced with Bohadschia vitiensis (Semper, 1868) ,[2] C ucumaria [illegible]datrix is not accepted, and replaced with Eupentacta fraudatrix (D'yakoov, Baranova & Savel'eva, 1958); [3] Cucumaria echinata is not accepted, and replaced with Pseudocnus echinatus (von Marenzeller,1882); [4]Neothyonidium magnum is accepted as Massinium magnum (Ludwig, 1882)*

*** the author mentioned the genus*

+The exact mass does not match with the reported formula, and structure

Table S3. Exact mass (*m/z*), molecular formula, retention time (RT), and Intensity signal (IntSig) of saponins, and sapogenins (aglycone parts) presented in the three sea cucumber genera *Holothuria, Bohadschia,* and *Actinopyga*

Molecular formula	Sample code	Exact mass (*m/z*)	*H. edulis*		*H. atra*		*H. hilla*		*H. whitmaei*		*B. vitiensis*		*Bohadschia sp.*		*B. argus*		*A. mauritiana*		*A. echinites*	
			RT	IntSig	RT	IntSig	RT	IntSig	RT	IntSig	RT	IntSig	RT	IntSig	RT	IntSig	RT	IntSig	RT	IntSig
$C_{29}H_{45}O_4$	M457T11.6	457.3318	11.63	184035	11.63	48457	11.65	19484	11.63	13514	11.63	78480	11.63	98646	11.65	47819	11.65	82074	11.65	94798
$C_{29}H_{45}O_4$	M457T12.1	457.3318	12.07	37295	12.07	3138	12.1	1861	12.07	1898	12.07	10742	12.07	15313	12.1	11938	12.1	5849	12.1	9126
$C_{29}H_{45}O_4$	M457T12.8	457.3318	12.8	11598	12.88	1333	-	-	-	-	12.82	1498	-	-	-	-	12.82	1034	-	-
$C_{29}H_{45}O_4$	M457T14.6	457.3318	14.58	68189	14.57	1694	14.52	6861	14.6	1555	14.6	12801	14.6	22148	14.6	5836	-	-	-	-
$C_{29}H_{45}O_4$	M457T14,7	457.3318	14.76	226972	14.76	14146	14.76	9550	14.76	3028	14.76	46297	14.76	61424			-	-	14.78	95012
$C_{29}H_{45}O_4$	M457T15	457.3318	14.98	206407	14.98	27010	15	3127	14.98	4266	14.98	37080	14.98	43936	15	24262	-	-	15	378109
$C_{30}H_{46}O_5$	M486T8.3	486.3345	-	-	-	-	-	-	-	-	-	-	-	-	8.27	13519	-	-	-	-
$C_{30}H_{46}O_5$	M486T8.7	486.3345	-	-	-	-	-	-	-	-	8.66	246	8.66	11359	8.66	92718	-	-	-	-
$C_{30}H_{46}O_5$	M486T8.8	486.3345	8.82	4398	8.82	2975	-	-	-	-	-	-	-	-	8.97	2541	8.82	8608	8.82	2560
$C_{30}H_{46}O_5$	M486T9.1	486.3345			9.11	13219	-	-	-	-	9.11	186	9.11	8764	9.13	46648	9.13	5966		
$C_{30}H_{46}O_5$	M486T9.3	486.3345	9.29	3674	9.27	29502	-	-	9.29	420	-	-	-	-	-	-	9.29	10541	9.29	2541
$C_{30}H_{46}O_5$	M486T9.6	486.3345	-	-	-	-	-	-	-	-	9.64	181	9.62	723	9.66	8180	-	-	-	-
$C_{30}H_{46}O_5$	M486T10	486.3345	-	-	-	-	9.74	773	9.74	534	-	-	-	-	-	-	10	16590	-	-
$C_{30}H_{46}O_5$	M486T10.4	486.3345	10.43	4413	10.3	2547			10.49	591	-	-	-	-	-	-	-	-	10.47	2314
$C_{30}H_{46}O_5$	M486T10.8	486.3345	-	-	10.77	46309	-	-	-	-	-	-	-	-	-	-	10.77	20257		
$C_{30}H_{46}O_5$	M486T11.8	486.3345	11.82	9471	-	-	-	-	-	-	-	-	-	-	-	-	-	-	11.79	2715
$C_{30}H_{48}O_4$	M472T9.9	472.3552	-	-	-	-	-	-	9.66	323	9.94	5288	9.94	13935	9.96	42508	-	-	-	-
$C_{30}H_{48}O_4$	M472T10.3	472.3552	10.3	3018	10.3	65	10.3	972	10.3	1078	10.3	59479	10.28	78073	10.28	79433	-	-	-	-
$C_{30}H_{48}O_4$	M472T10.5	472.3552	-	-	-	-	-	-	-	-	10.45	1091	10.49	5109	10.55	7531	-	-	-	-
$C_{30}H_{48}O_4$	M472T10.9	472.3552	10.88	2439	10.9	83	-	-	-	-	-	-	-	-	-	-	10.88	1154	10.88	2706
$C_{30}H_{48}O_4$	M472T11.4	472.3552	11.39	3304	-	-	-	-	-	-	-	-	-	-	-	-	11.39	4004	11.37	4258
$C_{30}H_{48}O_4$	M472T13.5	472.3552	-	-	-	-	-	-	-	-	-	-	-	-	-	-	13.44	23504	13.48	271
$C_{30}H_{42}O_5$	M482T6.8	482.3032	6.89	18464	-	-	-	-	-	-	-	-	-	-	-	-	-	-	-	-
$C_{30}H_{42}O_5$	M482T7.3	482.3032	-	-	-	-	-	-	-	-	-	-	-	-	7.29	2442	-	-	-	-
$C_{30}H_{42}O_5$	M482T8.4	482.3032	-	-	-	-	-	-	-	-	-	-	8.44	1136	8.42	4850	-	-	-	-
$C_{30}H_{42}O_5$	M482T8.5	482.3032	8.5	13170	8.5	50650	8.51	1081	-	-	-	-	-	-	-	-	8.5	6857	-	-
$C_{30}H_{42}O_5$	M8482T8.8	482.3032	-	-	-	-	8.8	5397	8.82	470	-	-	8.7	756	-	-	-	-	8.82	7571
$C_{30}H_{42}O_5$	M482T8.9	482.3032	8.97	38461	8.97	25652	8.95	39691	9	17036	-	-	8.91	487	8.93	2735	9	10770	9	8029
$C_{30}H_{42}O_5$	M482T9.1	482.3032	-	-	-	-	-	-	-	-	9.11	307	9.11	1722	-	-	-	-	-	-
$C_{30}H_{42}O_5$	M482T9.4	482.3032	9.38	12363	-	-	9.38	1943	9.38	1342	-	-	-	-	-	-	-	-	-	-
$C_{30}H_{42}O_5$	M482T9.6	482.3032	-	-	9.62	34737	-	-	-	-	-	-	-	-	9.62	1010	9.6	44740	-	-
$C_{30}H_{42}O_5$	M482T10.4	482.3032	-	-	10.34	14274	-	-	10.39	1409	-	-	-	-	-	-	10.3	20266	10.37	2467

$C_{30}H_{44}O_5$	M484T8.1	484.3189	-	-	-	-	-	-	-	-	8.1	665	8.1	4276	-	-	-	-	-	-
$C_{30}H_{44}O_5$	M484T8.4	484.3189	-	-	-	-	-	-	8.38	4902	8.46	1007	8.44	34833	-	-	-	-	-	-
$C_{30}H_{44}O_5$	M484T8.6	484.3189	8.62	2651	-	-	-	-	-	-	-	-	-	-	-	-	-	-	8.68	2266
$C_{30}H_{44}O_5$	M484T8.9	484.3189	8.97	296066	-	-	8.95	305644	-	-	-	-	8.91	5260	-	-	-	-	-	-
$C_{30}H_{44}O_5$	M484T9	484.3189	-	-	-	-	-	-	9	149532	9.02	642	-	-	-	-	9.32	176	9	78700
$C_{30}H_{44}O_5$	M484T10-4	484.3189	-	-	-	-	-	-	10.39	12701	-	-	-	-	-	-	-	-	10.37	24249
$C_{30}H_{44}O_5$	M484T11	484.3189	11.07	10806	-	-	-	-	-	-	-	-	-	-	-	-	-	-	-	-
$C_{30}H_{44}O_4$	M468T8.8	468.3239	8.82	9643	-	-	8.82	17494	8.82	626	-	-	-	-	-	-	8.82	24333	8.82	7273
$C_{30}H_{44}O_4$	M468T8.7	468.3239	-	-	-	-	-	-	-	-	8.66	449	8.66	11899	8.66	213137	-	-	-	-
$C_{30}H_{44}O_4$	M468T9.1	468.3239	-	-	9.11	16580			9.11	149	-	-	9.11	9601	9.13	75000	-	-	-	-
$C_{30}H_{44}O_4$	M468T9.3	468.3239	-	-	9.27	130539	9.29	22528	9.29	1240	-	-	-	-	-	-	9.29	31667	9.29	6290
$C_{30}H_{44}O_4$	M468T9.7	468.3239	-	-	-	-	9.74	3412	9.74	1942	-	-	-	-	-	-	-	-	-	-
$C_{30}H_{44}O_4$	M468T9.6	468.3239	-	-	-	-	-	-	-	-	9.64	876	9.62	7108	9.62	126607	-	-	-	-
$C_{30}H_{44}O_4$	M468T10	468.3239	-	-	-	-	-	-	-	-	-	-	-	-	-	-	10	33190	10.02	2394
$C_{30}H_{44}O_4$	M468T10.2	468.3239	-	-	-	-	-	-	-	-	10.19	1218	10.19	3587	-	-	-	-	-	-
$C_{30}H_{44}O_4$	M468T10.5	468.3239	10.45	114404	-	-	10.51	3631	10.49	10239	-	-	-	-	-	-	10.47	50322	10.47	41600
$C_{30}H_{44}O_4$	M468T10.8	468.3239			10.77	68089	-	-	-	-	-	-	-	-	-	-	10.77	29218	-	-
$C_{30}H_{44}O_4$	M468T11.1	468.3239	11.11	5257	-	-	-	-	-	-	-	-	-	-	-	-	-	-	11	25131
$C_{35}H_{52}O_8$	M600T7.12	600.3662	-	-	-	-	-	-	-	-	-	-	-	-	7.12	47562	-	-	-	-
$C_{35}H_{52}O_8$	M600T8.7	600.3662	-	-	-	-	8.87	425	-	-	-	-	8.66	2453	8.66	51372	-	-	-	-
$C_{35}H_{52}O_8$	M600T9.1	600.3662	-	-	9.11	4009			-	-	-	-	9.11	2160	9.13	20817	-	-	-	-
$C_{35}H_{52}O_8$	M600T9.3	600.3662	9.29	1175	9.27	26114	9.29	2709	9.29	127	-	-	-	-	-	-	9.29	4499	9.29	817
$C_{35}H_{52}O_8$	M600T9.6	600.3662	-	-	-	-	-	-	-	-	-	-	9.62	1181	9.62	28245	9.64	1383	-	-
$C_{35}H_{52}O_8$	M600T10.4	600.3662	10.45	21547	-	-	10.49	319	10.49	1243	10.49	629	10.49	1849	-	-	10.47	8604	10.47	6507
$C_{35}H_{52}O_8$	M600T10.7	600.3662	-		10.77	2731	-	-	-	-	-	-	-	-	-	-	10.77	1177		
$C_{35}H_{52}O_8$	M600T11	600.3662	-	-	-	-	-	-	-	-	-	-	-	-	-	-	11.07	1068	11.02	6497
$C_{35}H_{52}O_8$	M600T12.8	600.3662	-	-	-	-	-	-	-	-	-	-	-	-	-	-	12.82	3107	-	-
$C_{41}H_{66}O_{12}$	M750T9.9	750.4554	-	-	-	-	-	-	-	-	9.96	335	-	-	9.94	1104	-	-	-	-
$C_{41}H_{66}O_{12}$	M750T10.3	750.4554	-	-	-	-	10.3	296	10.3	234	10.3	20390	10.28	30195	10.32	30206	-	-	-	-
$C_{41}H_{66}O_{12}$	M750T10.9	750.4554	10.88	1822	-	-	-	-	-	-	-	-	-	-	10.92	1667	10.88	1058	10.88	2995
$C_{41}H_{66}O_{12}$	M750T11.3	750.4554	11.39	7058	-	-	-	-	-	-	11.28	42534	11.28	93291	11.3	53961	11.39	7813	11.37	9341
$C_{41}H_{66}O_{12}$	M750T13.5	750.4554	-	-	-	-	-	-	-	-	-	-	-	-	-	-	13.44	90887	13.46	3565
$C_{41}H_{64}O_{13}$	M764T8.7	764.4347	-	-	-	-	-	-	-	-	-	-	8.66	2511	8.66	25988	-	-	-	-
$C_{41}H_{64}O_{13}$	M764T9.1	764.4347	-	-	-	-	-	-	-	-	-	-	9.11	6055	9.13	38180	9.13	4040	-	-
$C_{41}H_{64}O_{13}$	M764T9.3	764.4347	9.29	6215	9.27	81762	9.29	16543	9.29	663	-	-	-	-	-	-	9.29	19563	9.29	4650
$C_{41}H_{64}O_{13}$	M764T9.4	764.4347	-	-	-	-	9.43	1478	-	-	9.4	559			9.4	6053	-	-	-	-
$C_{41}H_{64}O_{13}$	M764T9.7	764.4347	-	-	-	-	9.74	1393	9.74	798	9.74	833	9.74	4587	9.76	8769	-	-	-	-
$C_{41}H_{64}O_{13}$	M764T10.5	764.4347	10.43	11900	-	-	-	-	10.49	1662	-	-	-	-	-	-	-	-	10.47	5504
$C_{41}H_{64}O_{13}$	M764T10.8	764.4347	-	-	10.77	117043	-	-	-	-	10.75	588	-	-	-	-	10.77	51245	-	-

$C_{41}H_{64}O_{13}$	M764T12.2	764.4347	-	-	-	-	-	-	-	-	-	-	-	-	-	-	12.22	4931	12.2	3949
$C_{43}H_{66}O_{14}$	M806T9.3	806.4453	-	-	-	-	-	-	9.29	488	-	-	-	-	9.32	649	-	-	-	-
$C_{43}H_{66}O_{14}$	M806T9.7	806.4453	-	-	-	-	-	-	9.66	466	-	-	-	-	9.68	509	-	-	-	-
$C_{43}H_{66}O_{14}$	M806T9.8	806.4453	-	-	-	-	-	-	-	-	-	-	-	-	9.81	353	9.81	352	-	-
$C_{43}H_{66}O_{14}$	M806T10	806.4453	-	-	-	-	-	-	-	-	-	-	-	-	-	-	10.02	6544	10.02	1479
$C_{43}H_{66}O_{14}$	M806T11.2	806.4453	-	-	-	-	-	-	-	-	-	-	-	-	-	-	11.22	2751	-	-
$C_{41}H_{63}NaO_{16}S$	M866T10.8	866.3734	-	-	10.79	699	-	-	-	-	-	-	-	-	-	-	10.77	539	-	-
$C_{41}H_{63}NaO_{16}S$	M866T12.2	866.3734	-	-	-	-	-	-	-	-	-	-	-	-	-	-	12.2	567	12.22	532
$C_{41}H_{63}NaO_{16}S$	M866T15.1	866.3734	15.17	112	-	-	15.13	39	-	-	-	-	-	-	-	-	-	-	-	-
$C_{41}H_{63}NaO_{16}S$	M866T16.2	866.3734			-	-	-	-	-	-			16.23	71	16.23	82	-	-	-	-
$C_{41}H_{63}NaO_{17}S$	M882T10.4	882.3683	10.39	195	10.39	2033	-	-	10.41	238	10.32	48	10.32	62	-	-	10.37	730	10.37	700
$C_{41}H_{65}NaO_{17}S$	M882T14.2	882.384			-	-	-	-	-	-	14.17	83	14.27	71	-	-	-	-	-	-
$C_{41}H_{65}NaO_{17}S$	M882T15.8	882.384	15.84	57	-	-	15.6	73	15.74	79	-	-	-	-	-	-	-	-	-	-
$C_{41}H_{65}NaO_{16}S$	M868T12.9	868.3891	-	-	-	-	-	-	-	-	-	-	-	-	-	-	12.85	250	-	-
$C_{41}H_{65}NaO_{16}S$	M868T13.1	868.3891	-	-	-	-	-	-	13.18	48	-	-	-	-	13.19	52	13.1	1969	13.08	955
$C_{41}H_{65}NaO_{16}S$	M868T13	868.3891	-	-	-	-	-	-	-	-	-	-	-	-	-	-	-	-	-	-
$C_{40}H_{63}NaO_{17}S$	M870T8.1	870.3683	-	-	-	-	-	-	-	-			8.1	96	8.12	116	-	-	-	-
$C_{40}H_{63}NaO_{17}S$	M870T9.6	870.3683	-	-	9.6	580	-	-	-	-	-	-	-	-	-	-	9.6	742		
$C_{40}H_{63}NaO_{17}S$	M870T11.1	870.3683	-	-	-	-	-	-	-	-	-	-	-	-	-	-	11.16	268	11.16	187
$C_{40}H_{63}NaO_{17}S$	M870T13.8	870.3683	-	-	-	-	13.87	30	-	-	-	-	-	-	-	-	-	-	13.83	198
$C_{54}H_{86}O_{22}$	M1086T9.7	1086.561	-	-	9.11	251	-	-	-	-	-	-	9.76	413	9.74	831	-	-	-	-
$C_{54}H_{86}O_{22}$	M1086T10.7	1086.561	10.64	1929			-	-	-	-	10.75	1093	10.75	1276	10.28	630	-	-	10.64	306
$C_{54}H_{86}O_{22}$	M1086T11.1	1086.561	11.11	31369	11.16	197	-	-	11.16	71	-	-	-	-	-	-	11.09	28112	11.07	64326
$C_{54}H_{88}O_{22}$	M1088T10.3	1088.5767	-	-	-	-	-	-			10.28	382	10.28	1109	-	-	-	-	-	-
$C_{54}H_{88}O_{22}$	M1088T10.9	1088.5767	-	-	-	-	10.92	29	-	-	-	-	-	-	10.9	116	10.9	386	10.9	3968
$C_{54}H_{88}O_{22}$	M1088T11.4	1088.5767	11.39	1014	-	-	-	-	-	-	-	-	-	-	10.35	739	11.39	1952	11.37	2345
$C_{54}H_{86}O_{23}$	M1102T9.1	1102.5559	-	-	9.11	27206	-	-	-	-	-	-	9.11	6116	9.13	171722	9.13	8034	-	-
$C_{54}H_{86}O_{23}$	M1102T9.3	1102.5559	-	-	9.29	14417	9.29	2229	-	-	-	-	-	-	-	-	9.29	2265	9.29	702
$C_{54}H_{86}O_{23}$	M1102T10.5	1102.5559	10.47	16517	10.49	2917			10.49	2319	-	-	-	-	-	-	10.45	8906	10.47	8951
$C_{54}H_{86}O_{23}$	M1102T11.8	1102.5559	11.82	2111	-	-	-	-	-	-	-	-	-	-	-	-	-	-	11.79	884
$C_{53}H_{84}O_{24}$	M1104T8.6	1104.5353	8.64	41	-	-	-	-	-	-	-	-			8.64	348	8.68	291	8.68	347
$C_{53}H_{84}O_{24}$	M1104T8.4	1104.5353	-	-	-	-	-	-	-	-	-	-	8.44	149	8.52	1486	8.59	185	-	-
$C_{54}H_{88}O_{24}$	M1104T10	1104.5716	-	-	-	-	-	-	-	-	-	-	9.94	173	9.96	300	-	-	-	-
$C_{54}H_{88}O_{24}$	M1104T10.7	1104.5716	-	-	-	-	-	-	-	-	-	-	-	-	-	-	10.71	244	10.71	933
$C_{54}H_{88}O_{24}$	M1104T11.1	1104.5716	11.11	19192	11.16	675	11.16	192	11.16	396	11.16	175	11.16	219	11.16	518	11.09	13705	11.09	24662
$C_{54}H_{84}O_{24}$	M1116T9.4	1116.5353	9.38	7544	9.38	1489	9.4	532	9.38	241	-	-	9.38	226	-	-	9.38	271	-	-
$C_{54}H_{86}O_{24}$	M1118T6.9	1118.5509	-	-	-	-	-	-	-	-	-	-	-	-	6.99	12629	-	-	-	-

$C_{54}H_{86}O_{24}$	M1118T8.7	1118.5509	-	-	-	-	-	-	-	-	-	-	8.66	1302	8.68	26858	-	-	-	-
$C_{54}H_{86}O_{24}$	M1118T8.9	1118.5509	8.93	4843	8.95	3834	8.93	4516	8.91	1079	8.91	243	8.91	5919	8.93	153956	8.93	61994	-	-
$C_{54}H_{86}O_{24}$	M1118T9	1118.5509	9.02	9537	9.02	5741	9.04	10800	9.02	14126	-	-	-	-	-	-	-	-	9.02	8791
$C_{54}H_{88}O_{23}$	M1120T8.8	1120.5666	8.82	690	8.8	264	8.82	952	-	-	-	-	-	-	-	-	8.78	2578	8.82	494
$C_{56}H_{88}O_{24}$	M1144T8.5	1144.5665	-	-	8.48	261	-	-	-	-	-	-	-	-	8.5	532	-	-	-	-
$C_{56}H_{88}O_{24}$	M1144T9.8	1144.5665	-	-	-	-	-	-	-	-	-	-	-	-	9.81	2222	9.81	450	-	-
$C_{56}H_{88}O_{24}$	M1144T10	1144.5665	-	-	-	-	-	-	-	-	-	-	-	-	-	-	10.02	2021	10.04	445
$C_{54}H_{88}O_{25}S$	M1166T11.2	1166.5178	11.11	544	11.16	40	-	-	-	-	-	-	-	-	-	-	11.16	649	11.02	194
$C_{54}H_{88}O_{25}S$	M1168T8.5	1168.5335					-	-	-	-	-	-	8.48	477	8.5	327	-	-	-	-
$C_{56}H_{88}O_{26}$	M1176T8.5	1176.5563	8.5	1397	8.5	3591	-	-	-	-	-	-	-	-	8.42	9251	-	-	-	-
$C_{56}H_{88}O_{26}$	M1176T8.1	1176.5563	-	-	-	-	-	-	-	-	8.1	143	8.1	1327	8.1	15995	-	-	-	-
$C_{56}H_{88}O_{26}$	M1176T11.7	1176.5563	-	-	-	-	-	-	-	-	-	-	-	-	-	-	11.65	3144	11.65	467
$C_{55}H_{85}NaO_{25}S$	M1200T8.8	1200.4998	8.82	943	-	-	8.82	2291	8.8	59	-	-	-	-	8.82	415	-	-	-	-
$C_{55}H_{85}NaO_{25}S$	M1200T11.3	1200.4998	-	-	-	-	-	-	-	-	11.3	52	11.3	61	-	-	11.16	4780	11.26	389
$C_{54}H_{85}NaO_{26}S$	M1204T10.5	1204.4947	10.49	149	-	-	-	-	-	-	-	-	-	-	-	-	10.49	689	10.51	112
$C_{54}H_{85}NaO_{27}S$	M1220T9	1220.4896	9.06	153	-	-	-	-	9.06	115	-	-	-	-	-	-	9.02	197	9.02	121
$C_{54}H_{85}NaO_{27}S$	M1220T10.8	1220.4896	-	-	-	-	-	-	-	-	10.77	56	10.77	75	-	-	-	-	-	-
$C_{54}H_{87}NaO_{26}S$	M1206T9.7	1206.5104	-	-	-	-	-	-	-	-	9.74	35	9.76	183	8.12	220	-	-	-	-
$C_{54}H_{87}NaO_{26}S$	M1206T10.4	1206.5104	10.43	30	-	-	-	-	-	-	-	-	-	-	10.13	161	-	-	-	-
$C_{54}H_{87}NaO_{26}S$	M1206T11.2	1206.5104	-	-	11.14	28	11.18	21	-	-	-	-	11.3	168	11.84	122	11.16	151	11.14	313
$C_{53}H_{85}NaO_{27}S$	M1206T9.1	1206.474	-	-	9.13	120		-	-	-	-	-	-	-	9.13	744	-	-	-	-
$C_{53}H_{85}NaO_{27}S$	M1206T10.5	1206.474	-	-	10.49	127	10.49	38	10.49	119	-	-	-	-	-	-	-	-	-	-
$C_{53}H_{85}NaO_{27}S$	M1206T11.1	1206.474	11.11	935	-	-	-	-	-	-	-	-	-	-	-	-	11.11	1791	11.07	3125
$C_{53}H_{85}NaO_{27}S$	M1206T11.4	1206.474	11.37	688	-	-	-	-	-	-	-	-	-	-	-	-	11.39	4763	11.37	5440
$C_{53}H_{84}NaO_{27}S$	M1207T8.1	1207.4818	-	-	-	-	-	-	-	-	8.08	75	8.1	307	8.12	1213	-	-	-	-
$C_{53}H_{84}NaO_{27}S$	M1207T8.5	1207.4818	-	-	8.48	612	-	-	-	-	-	-	-	-	-	-	-	-	-	-
$C_{53}H_{84}NaO_{27}S$	M1207T11.1	1207.4818	11.11	853	-	-	-	-	-	-	-	-	-	-	-	-	11.07	124	11.07	169
$C_{53}H_{84}NaO_{27}S$	M1207T11.4	1207.4818	11.39	1684	-	-	-	-	-	-	-	-	-	-	-	-	11.37	818	11.41	251
$C_{55}H_{83}NaO_{26}S$	M1214T8.5	1214.4791	-	-	8.46	1607	-	-	-	-	-	-	8.48	2371	8.5	1726	-	-	-	-
$C_{54}H_{85}NaO_{27}S$	M1220T9	1220.4896	9	149	8.95	232	-	-	-	-	-	-	-	-	-	-	-	-	-	-
$C_{54}H_{85}NaO_{27}S$	M1220T9.1	1220.4896	9.06	185	-	-	9.04	846	9.04	900	-	-	-	-	-	-	9.02	197	9.02	121
$C_{54}H_{87}NaO_{27}S$	M1222T10.7	1222.5053	-	-	-	-	-	-	-	-	10.75	124	10.75	101	10.15	185	-	-	-	-
$C_{53}H_{80}O_{28}S_2$	M1228T8.8	1228.4277	-	-	-	-	-	-	-	-	-	-	8.48	81	8.82	87	8.(2	975	-	-
$C_{53}H_{80}O_{28}S_2$	M1228T9.4	1228.4277	9.38	23499	9.38	4429	9.4	2087	9.38	1069	-	-	-	-	-	-	-	-	-	-
$C_{55}H_{83}NaO_{27}S$	M1230T8.1	1230.474	-	-	-	-	-	-	-	-	8.1	155	8.1	459	8.12	1408	8.15	170	-	-
$C_{55}H_{83}NaO_{27}S$	M1230T8.4	1230.474	8.52	90	8.4	287	-	-	-	-	-	-	8.44	377	8.42	194	-	-	-	-
$C_{55}H_{83}NaO_{27}S$	M1230T11	1230.474	-	-	-	-	9.43	107	-	-	-	-	-	-	-	-	10.94	115	11.37	121

$C_{54}H_{85}NaO_{28}S$	M1236T8.5	1236.4846	8.57	26	-	-	-	-	-	-	-	-	8.44	127	8.48	147	-	-	-	-
$C_{60}H_{94}O_{27}$	M1246T8.7	1246.5982			-	-	-	-	-	-	-	-	8.66	123	8.68	14019	-	-	-	-
$C_{60}H_{94}O_{27}$	M1246T9	1246.5982	8.97	355	8.97	229	8.95	135	9	159	-	-	-	-	-	-	9	108	-	-
$C_{60}H_{94}O_{27}$	M1246T9.4	1246.5982	-	-	-	-	9.4	63	-	-	-	-	-	-	9.47	1828	-	-	-	-
$C_{60}H_{94}O_{27}$	M1246T9.6	1246.5982	-	-	-	-	-	-	-	-	-	-	9.62	246	9.66	10612	-	-	-	-
$C_{60}H_{94}O_{27}$	M1246T10.7	1246.5982	-	-	-	-	-	-	-	-	10.75	152	10.75	232	-	-	-	-	-	-
$C_{60}H_{96}O_{27}$	M1248T9.4	1248.6139	-	-	-	-	-	-	-	-	-	-	-	-	9.45	4543	-	-	-	-
$C_{60}H_{96}O_{27}$	M1248T9.8	1248.6139	-	-	-	-	-	-	-	-	9.74	143	9.76	655	9.78	12306	-	-	-	-
$C_{60}H_{96}O_{27}$	M1248T10.8	1248.6139	-	-	-	-	-	-	-	-	10.75	1633	10.75	1577	-	-	-	-	-	-
$C_{60}H_{96}O_{27}$	M1248T11.3	1248.6139	-	-	-	-	-	-	-	-	11.28	562	11.33	117	-	-	-	-	-	-
$C_{60}H_{96}O_{27}$	M1248T11	1248.6139	-	-	-	-	-	-	-	-	-	-	-	-	-	-	11.09	110	11.04	254
$C_{60}H_{98}O_{27}$	M1250T10.3	1250.6295	10.3	105	-	-	-	-	-	-	10.3	2014	10.35	3031	10.35	2758	-	-	-	-
$C_{53}H_{82}Na_2O_{27}S_2$	M1260T10	1260.428	-	-	-	-	-	-	-	-	-	-	-	-	-	-	10.02	1246	-	-
$C_{53}H_{82}Na_2O_{27}S_2$	M1260T9	1260.428	-	-	9.02	249	9	90	9	50	-	-	-	-	-	-	-	-	-	-
$C_{60}H_{96}O_{28}$	M1264T8.6	1264.6088	-	-	-	-	-	-	-	-	-	-	8.66	2456	8.68	222601	8.64	127	-	-
$C_{60}H_{96}O_{28}$	M1264T9.5	1264.6088	-	-	-	-	9.43	1568	-	-	-	-	-	-	9.49	14169	-	-	-	-
$C_{60}H_{96}O_{28}$	M1264T9.7	1264.6088	-	-	-	-	-	-	-	-	9.64	183	9.64	799	9.66	129520	-	-	-	-
$C_{60}H_{96}O_{28}$	M1264T8.7	1264.6088	-	-	-	-	-	-	-	-			8.66	2456	8.68	222601	8.64	127	-	-
$C_{60}H_{96}O_{28}$	M1264T9.6	1264.6088	-	-	-	-	9.43	1568	-	-	9.64	183	9.64	799	9.66	129520	-	-	-	-
$C_{60}H_{98}O_{28}$	M1266T10.7	1266.6245	-	-	10.71	47	-	-	-	-	10.75	56			10.17	1017	-	-	10.15	106
$C_{60}H_{96}O_{29}$	M1280T8.4	1280.6037	-	-	-	-	-	-	-	-	8.44	118	8.44	16409	8.44	390904	8.44	476	-	-
$C_{62}H_{100}O_{29}$	M1308T9.7	1308.635			-	-	-	-	-	-	-	-	9.72	32	9.74	158	-	-	-	-
$C_{62}H_{100}O_{29}$	M1308T11.6	1308.635	11.61	54	-	-	-	-	-	-	-	-	-	-	-		11.61	142	11.58	61
$C_{59}H_{90}O_{32}S_2$	M1374T9	1374.4856	9	422	9.25	162	8.97	280	9	347	-	-	-	-	9.47	209	9.29	224	9	106
$C_{66}H_{104}O_{31}$	M1392T8.7	1392.6561	-	-	-	-	-	-	-	-	-	-	-	-	8.66	7963	-	-	-	-
$C_{66}H_{104}O_{31}$	M1392T9.4	1392.6561	-	-	9.27	73	-	-	-	-	-	-	-	-	9.47	134321	-	-	-	-
$C_{66}H_{104}O_{31}$	M1392T9.6	1392.6561	-	-	-	-	-	-	-	-	-	-	9.62	1276	9.62	23799	-	-	-	-
$C_{66}H_{104}O_{31}$	M1392T9.8	1392.6561	-	-	-	-	-	-	-	-	-	-	9.74	8590	9.76	7551	-	-	-	-
$C_{66}H_{104}O_{31}$	M1392T10.7	1392.6561	10.43	123	-	-	-	-	-	-	10.75	5008	10.73	1938	-	-	-	-	-	-
$C_{67}H_{110}O_{31}$	M1410T10.3	1410.703			-	-	-	-	-	-			10.28	9506	10.32	9254	-	-	-	-
$C_{67}H_{110}O_{31}$	M1410T11.3	1410.703	11.28	173	-	-	-	-	11.28	41	11.3	270185	11.3	403627	11.3	258252	-	-	-	-
$C_{67}H_{110}O_{31}$	M1410T13	1410.703	-	-	-	-	-	-	-	-	-	-	-	-	-	-	12.99	1307	13.01	1213
$C_{67}H_{106}O_{32}$	M1422T9.6	1422.6667	-	-	-	-	-	-	-	-	-	-	9.4	1074	9.4	66846	-	-	-	-
$C_{67}H_{106}O_{32}$	M1422T9.8	1422.6667	-	-	-	-	-	-	-	-	-	-	9.74	8590	9.76	7551	-	-	-	-
$C_{67}H_{108}O_{32}$	M1424T9.6	1424.6823	-	-	-	-	-	-	-	-	-	-	9.62	20084	-	-	9.62	87900	-	-
$C_{67}H_{108}O_{32}$	M1424T9.4	1424.6823	-	-	-	-	9.4	25	-	-	-	-	-	-	9.45	18099	-	-	-	-
$C_{67}H_{108}O_{32}$	M1424T9.8	1424.6823	-	-	-	-	-	-	-	-	9.74	8846	9.76	175639	9.76	398783	-	-	-	-

$C_{67}H_{108}O_{32}$	M1424T10.8	1424.6823	-	-	-	-	-	-	-	-	10.75	146286	10.75	170102	-	-	-	-	-	-
$C_{67}H_{110}O_{32}$	M1426T9.8	1426.698	-	-	-	-	-	-	-	-	-	-	9.76	24745	9.72	22319	-	-	-	-
$C_{67}H_{110}O_{32}$	M1426T10.3	1426.698	10.3	2675	10.3	17	10.28	45	10.28	73	10.3	253640	10.3	349016	10.32	500577	-	-	10.3	99
$C_{67}H_{110}O_{32}$	M1426T10.8	1426.698	-	-	-	-	-	-	-	-	10.75	44754	10.75	46554	-	-	-	-	-	-
$C_{68}H_{110}O_{32}$	M1438T10.2	1438.698	-	-	-	-	-	-	10.17	1277	-	-	-	-	10.17	13230	-	-	-	-
$C_{67}H_{108}O_{33}$	M1440T7.12	1440.6772	-	-	-	-	-	-	-	-	-	-	-	-	7.12	226118	-	-	-	-
$C_{67}H_{108}O_{33}$	M1440T7.4	1440.6772	-	-	-	-	-	-	-	-	-	-	-	-	7.38	128454	-	-	-	-
$C_{67}H_{108}O_{33}$	M1440T8.7	1440.6772	-	-	-	-	-	-	-	-	-	-	8.66	4178	8.66	198297	8.19	108	-	-
$C_{67}H_{108}O_{33}$	M1440T9.6	1440.6772	-	-	-	-	-	-	-	-	-	-	9.62	20073	9.64	360514	-	-	-	-
$C_{67}H_{110}O_{33}$	M1442T7.3	1442.6929	-	-	-	-	-	-	-	-	-	-	-	-	7.38	17805	-	-	-	-
$C_{67}H_{110}O_{33}$	M1442T8.7	1442.6929	-	-	-	-	-	-	-	-	-	-	-	-	8.66	57137	-	-	-	-
$C_{67}H_{110}O_{33}$	M1442T9.3	1442.6929	9.36	33	9.31	28	-		-	-	-	-		-	-	-	-	-	-	-
$C_{67}H_{110}O_{33}$	M1442T9.9	1442.6929	-	-	-	-	-	-	-	-	9.94	1487	9.94	22617	9.96	162540	-	-	-	-
$C_{67}H_{110}O_{33}$	M1442T10.1	1442.6929	-	-	-	-	-	-	-	-	-		10.13	1217	10.13	154000	-	-	-	-
$C_{68}H_{110}O_{33}$	M1454T9.8	1454.6929	-	-	-	-	-	-	9.81	513	-	-	-	-	9.81	7674	-	-	-	-

Table S4. Exact mass (*m/z*), molecular formula, retention time (RT in minutes), and Intensity signal of saponins presented in isolated fractions of *B. argus*

Exact mass (*m/z*)	Compound code	Fr.1		Fr.2		Fr.3		Fr.4	
		RT	Intensity signal	RT	Intensity signal	RT	Intensity signal	RT	Intensity signal
468.3239	$C_{30}H_{44}O_4$	7.33	28424	8.62	123481	10	93	9.08	421
468.3239	$C_{30}H_{44}O_4$	7.12	15096	8.44	24853	10.21	66	10.15	470
600.3662	$C_{35}H_{52}O_8$	7.33	23077	8.44	33649	10.21	19	10.13	245
600.3662	$C_{35}H_{52}O_8$	7.12	19415	8.62	24716	-	-	10	119
1116.535	$C_{54}H_{84}O_{24}$	7.42	557	8.06	1177	10.58	54	9.34	79
1118.551	$C_{54}H_{86}O_{24}$	7.42	16946	8.64	20184	10.62	59	8.89	28
1118.551	$C_{54}H_{86}O_{24}$	7.59	11152	-	-	-	-	-	-
1120.567	$C_{54}H_{88}O_{23}$	7.74	146	8.4	142	13.25	12	5.83	28
1144.567	$C_{56}H_{88}O_{24}$	7.4	36	8.62	101	10.62	15	10.25	74
1214.479	$C_{55}H_{83}NaO_{26}S$	8.03	1105	8.44	5509	10.6	75	8.25	22
1264.609	$C_{60}H_{96}O_{28}$	7.33	255	8.62	18308	10.35	15	10.92	105
1280.604	$C_{60}H_{96}O_{29}$	5.43	68	8.4	59158	10.58	58	12.07	27
1424.682	$C_{67}H_{108}O_{32}$	7.93	4324	8.64	51	10.35	4450	10.28	513
1426.698	$C_{67}H_{110}O_{32}$	7.97	61	8.48	105	10.39	465477	10.31	139189
1438.698	$C_{54}H_{84}O_{24}$	7.65	28	8.46	33	10.71	30	10.28	51
1422.667	$C_{66}H_{104}O_{31}$	7.33	670	8.62	1388	10.28	501	10.25	175
1422.667	$C_{66}H_{104}O_{31}$	7.93	316	8.46	180	10.37	242	-	-
1440.677	$C_{67}H_{108}O_{33}$	7.33	32702	8.62	113676	10.41	277	10.25	84
1440.677	$C_{67}H_{108}O_{33}$	7.93	9991	-	-	-	-	-	-
1442.693	$C_{67}H_{110}O_{33}$	7.33	6165	8.52	19	10.66	115	10.06	506
1442.693	$C_{67}H_{110}O_{33}$	7.48	3920	-	-	10.41	-	9.9	687
1454.693	$C_{68}H_{110}O_{33}$	7.69	28	8.36	27	10.66	142955	9.96	26
1454.693	$C_{68}H_{110}O_{33}$	-	-	-	-	10.75	124314	-	-

Figures:

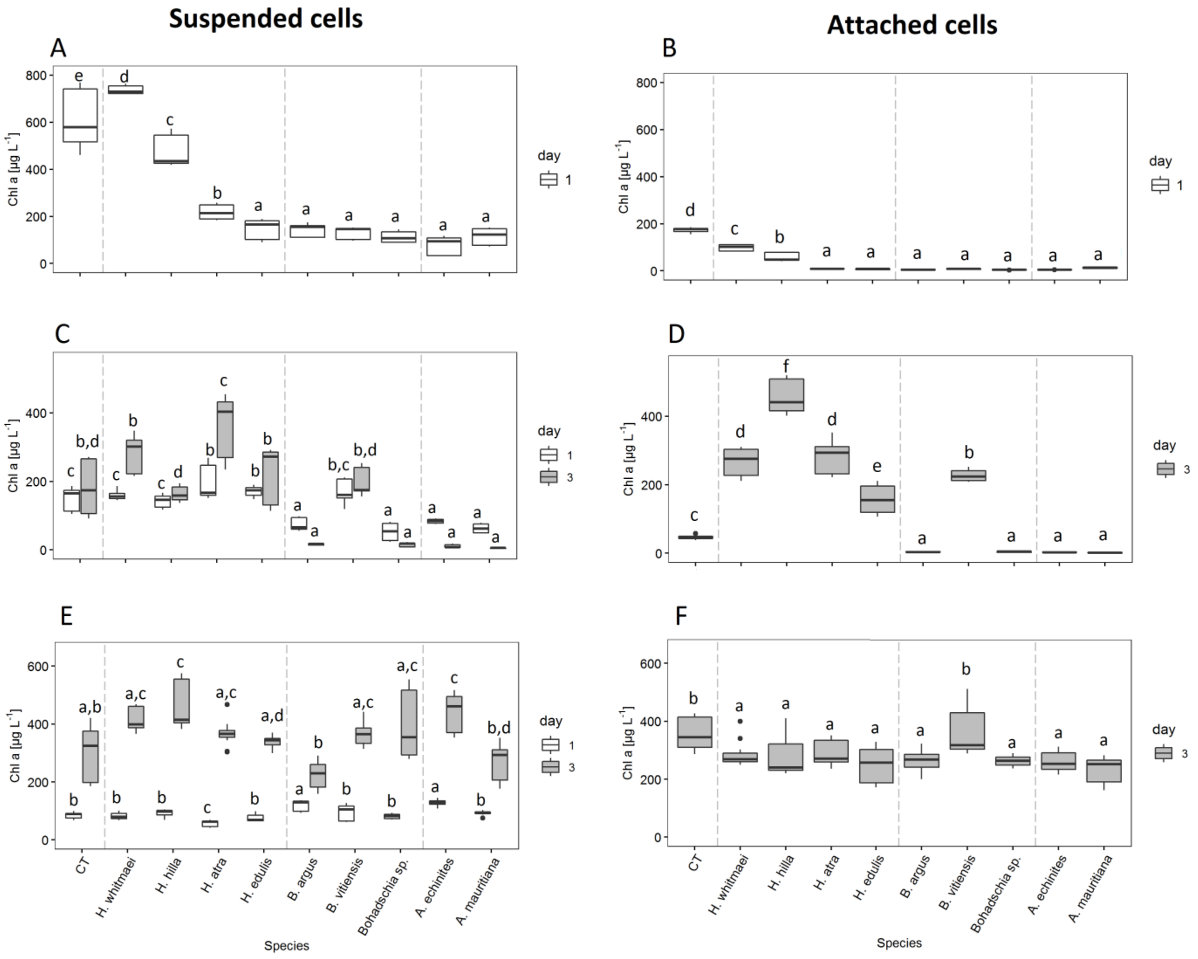

Figure S1(A-F). *Chl a* concentrations in the suspended cells in water after incubation of *C. closterium* with different concentrations of sea cucumbers extracts (**A**=150 µg mL^{-1}; **C**= 15 µg mL^{-1}; **E**= 1.5 µg mL^{-1}) and of *C. closterium* attached to the flask surface (**B**=150 µg mL^{-1}; **D**= 15 µg mL^{-1}; **F**=1.5 µg mL^{-1}). Dashed lines separate different genera of sea cucumbers (*Holothuria*, *Bohadschia*, *Actinopyga*). CT=Control. [a-e] indicate significance levels according to Post Hoc test.

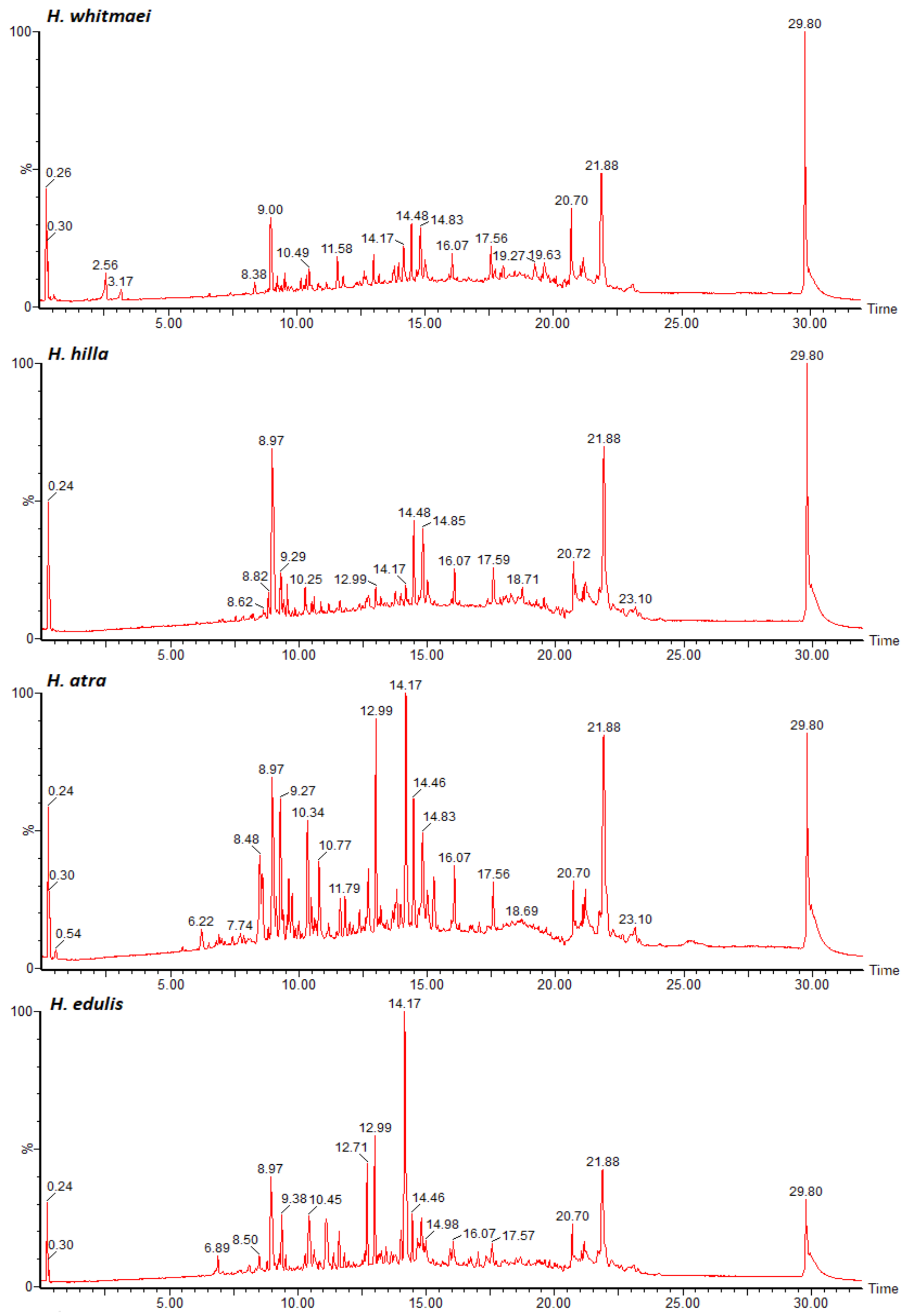

Figure S2. LC/MS spectra of the crude extracts of genus *Holothuria* (Y-axis relative intensity in % of maximum peak, x-axis retention time in minutes).

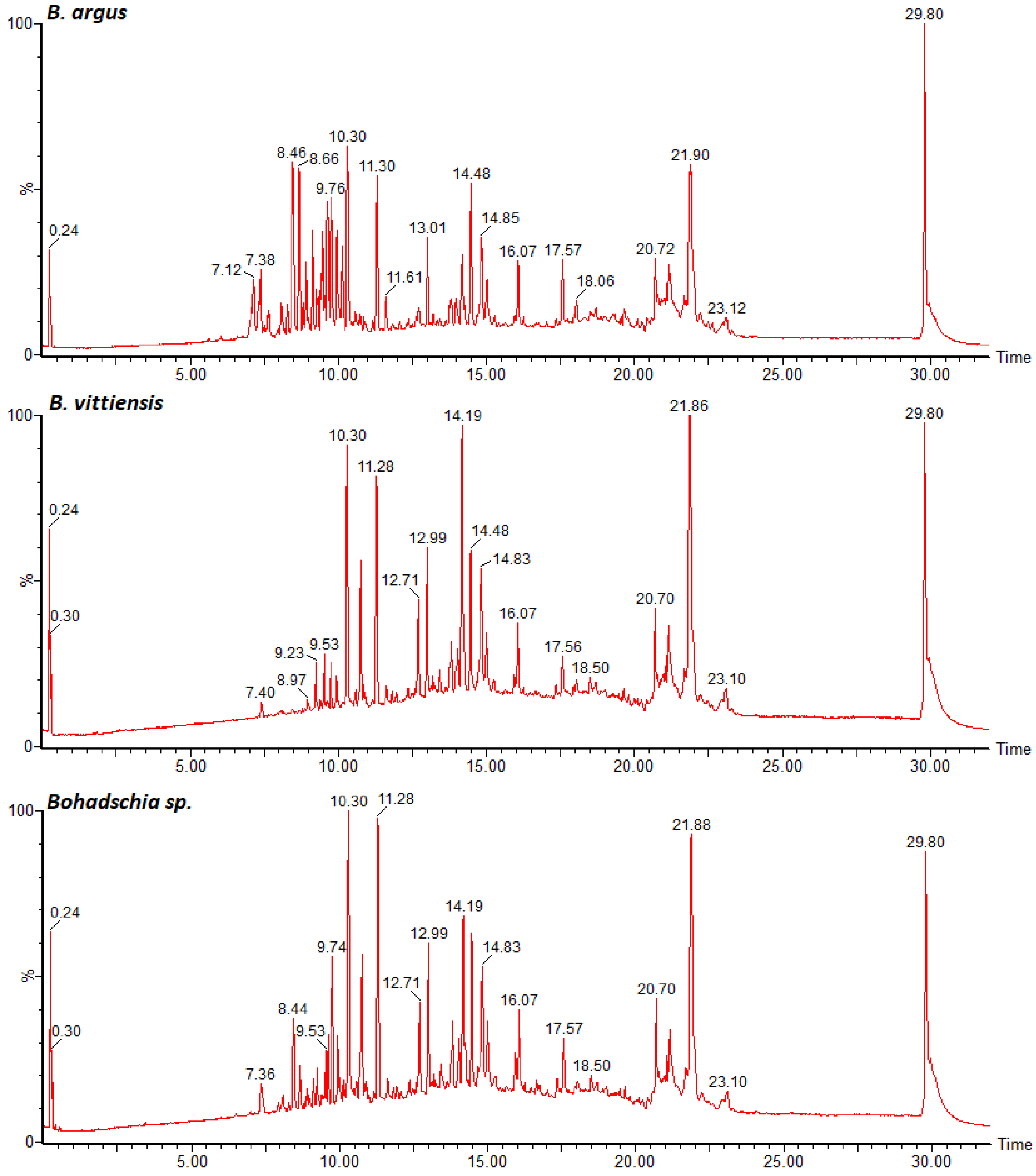

Figure S3. LC/MS spectra of the crude extracts of genus *Bohadschia* (Y-axis relative intensity in % of maximum peak, x-axis retention time in minutes).

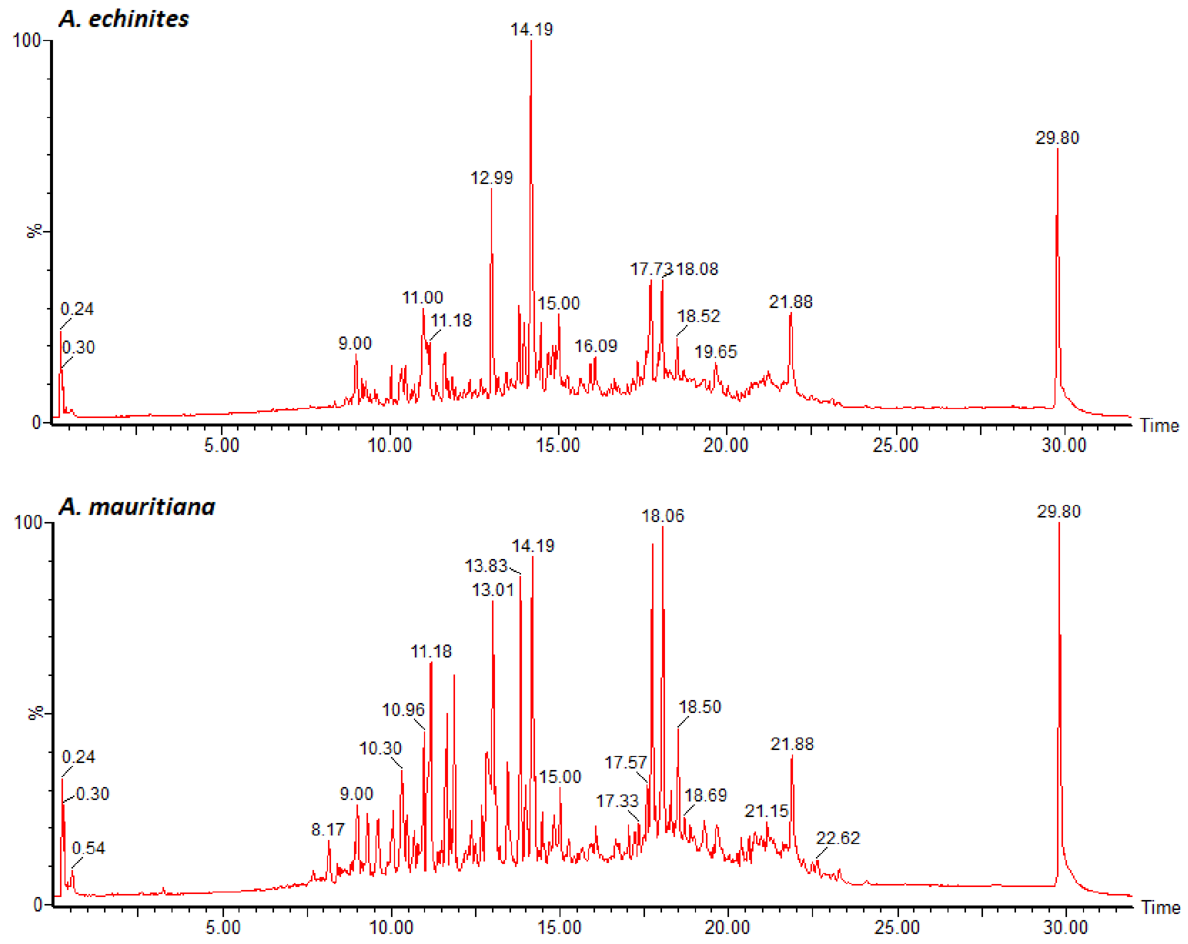

Figure S4. LC/MS spectra of the crude extracts of genus *Actinopyga* (Y-axis relative intensity in % of maximum peak, x-axis retention time in minutes).

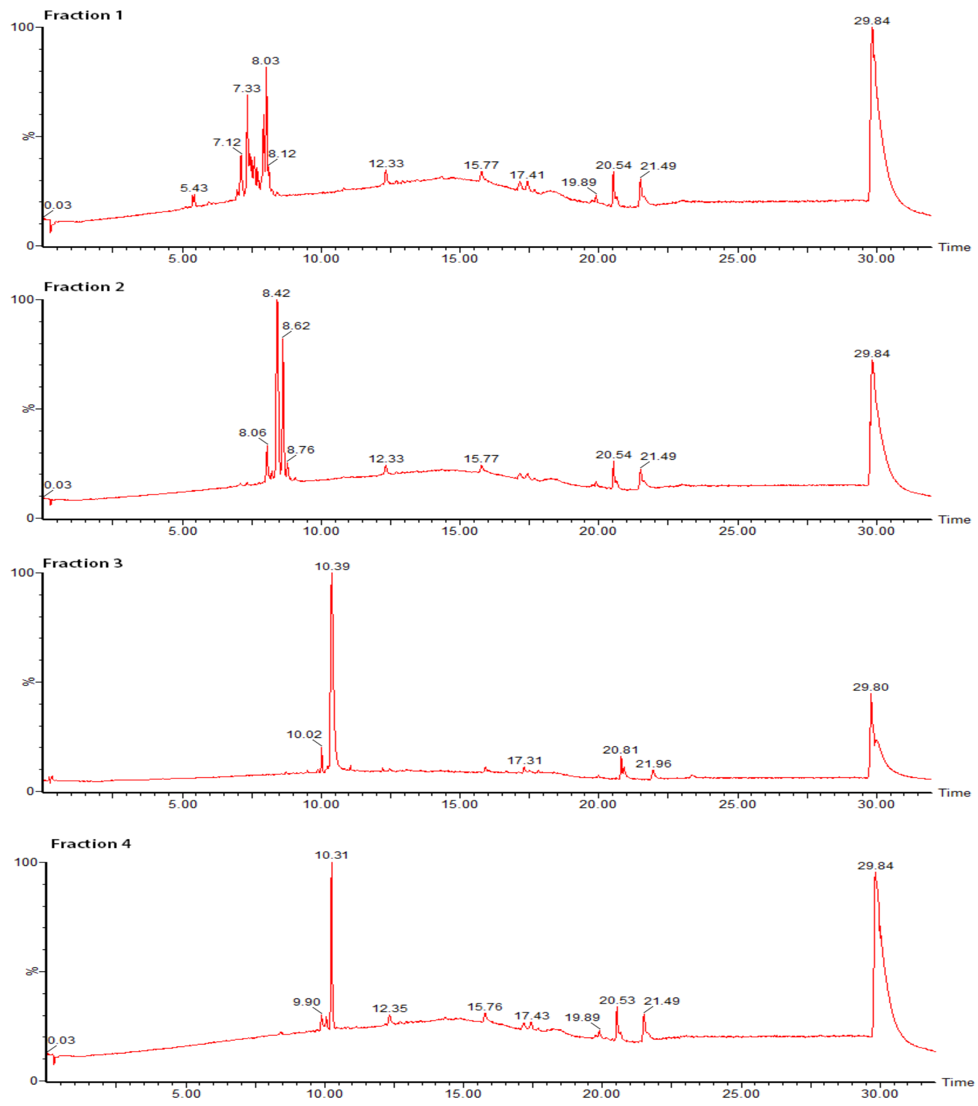

Figure S5. LC/MS spectra of fractions isolated from *B. argus* (see **Table S4**; Y-axis relative intensity in % of maximum peak, x-axis retention time in minutes).

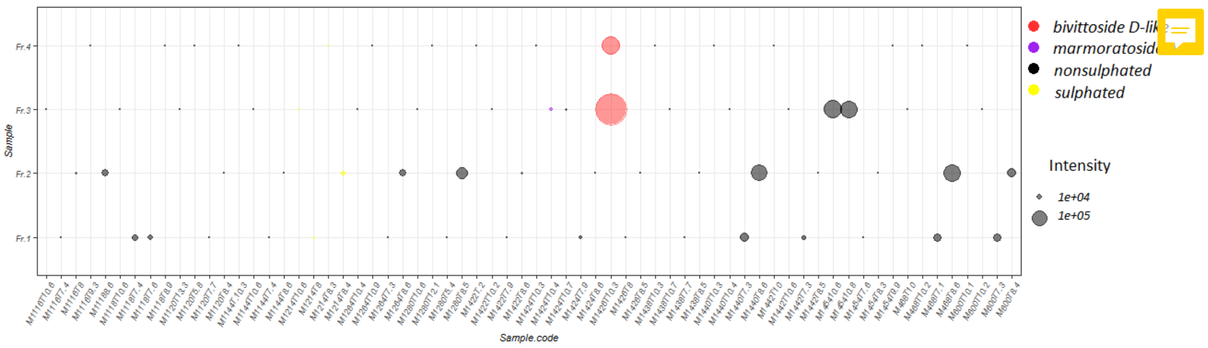

Figure S6 Identified saponins species presented in different fractions isolated from *B. argus.* The red color referred to the presence of a semi-purified saponin species (i.e. *bivittoside D-like* at m/z 1426.698). Size of bubbles represented the peak area of the molecules obtained from LC/MS analysis.

References from Table S1:

Afiyatullov SS, Ya. Tishchenko L, A. Stonik V, Kalinovsky A, B. Elyakov G (1985) Structure of cucumarioside G 1 - A new triterpene glycoside from the holothurian Cucumaria fraudatrix. Chem Nat Compd - CHEM NAT COMPD 21:228–232 . doi: 10.1007/BF00714918

Avilov SA, Antonov AS, Drozdova OA, Kalinin VI, Kalinovsky AI, Stonik VA, Riguera R, Lenis LA, Jiménez C (2000) Triterpene glycosides from the Far-Eastern sea cucumber Pentamera calcigera. 1. Monosulfated glycosides and cytotoxicity of their unsulfated derivatives. J Nat Prod 63:65–71 . doi: 10.1021/np9903447

Avilov SA, Silchenko AS, Antonov AS, Kalinin VI, Kalinovsky AI, Smirnov A V, Dmitrenok PS, Evtushenko E V, Fedorov SN, Savina AS, Shubina LK, Stonik VA (2008) Synaptosides A and A1 , triterpene glycosides from the sea cucumber Synapta maculata containing 3-O-methylglucuronic acid and their cytotoxic activity against tumor cells. J Nat Prod 71:525–531

Avilov SA, Stonik VA (1988) New triterpene glycosides from the holothurian Cladolabes sp. 764–765

Bahrami Y, Franco C (2015) Structure Elucidation of New Acetylated Saponins, Lessoniosides A, B, C, D, and E, and Non-Acetylated Saponins, Lessoniosides F and G, from the Viscera of the Sea Cucumber Holothuria lessoni. Mar Drugs 13:597–617 . doi: 10.3390/md13010597

Bahrami Y, Franco CMM, Benkendorff K (2016) Acetylated triterpene glycosides and their biological activity from holothuroidea reported in the past six decades. Mar Drugs 14:1–38 . doi: 10.3390/md14080147

Bahrami Y, Zhang W, Chataway T, Franco C (2014a) Structural elucidation of novel saponins in the sea cucumber Holothuria lessoni. Mar Drugs 12:4439–4473 . doi: 10.3390/md12084439

Bahrami Y, Zhang W, Franco C (2014b) Discovery of novel saponins from the viscera of the sea cucumber Holothuria lessoni. Mar Drugs 12:2633–2667 . doi: 10.3390/md12052633

Bahrami Y, Zhang W, M M Franco C (2018) Distribution of Saponins in the Sea Cucumber Holothuria lessoni; the Body Wall Versus the Viscera, and Their Biological Activities. Mar Drugs 16: . doi: 10.3390/md16110423

Bhatnagar S, Dudouet B, Ahond A, Poupat C, Thoison O, Clastres A, Laurent D, Potier P (1985) Invertebres marins du lagon Neocaledonien IV. Saponines et sapogenines d'une holothurie, Actinopyga flammea. Bull Soc Chim Fr 124–129

Cuong NX, Vien LT, Hoang L, Hanh TTH, Thao DT, Thanh N Van, Nam NH, Thung DC, Kiem P Van, Minh C Van (2017) Cytotoxic triterpene diglycosides from the sea cucumber Stichopus horrens. Bioorg Med Chem Lett 27:2939–2942 . doi: https://doi.org/10.1016/j.bmcl.2017.05.003

Drozdova OA, Avilov SA, Kalinin VI, Kalinovsky AI, Stonik VA, Riguera R, Jiménez C (1997) Cytotoxic triterpene glycosides from far-eastern sea cucumbers belonging to the genus Cucumaria. Liebigs Ann 2351–2356 . doi: 10.1002/jlac.199719971125

Elbandy M, Rho JR, Afifi R (2014) Analysis of saponins as bioactive zoochemicals from the marine functional food sea cucumber Bohadschia cousteaui. Eur Food Res Technol 238:937–955 . doi: 10.1007/s00217-014-2171-6

Elyakov GB (1983) Structure of holotoxin A1 (stichoposide A) a basic triterpene glycoside of the Pacific sea cucumber Stichopus japonicus Selenka. CA 98: 176525n. Bioorg Khim 9:280–282

Hal Dang N, Van Thanh N, Van Kiem P, Mai Huong L, Van Minh C, Ho Kim Y (2007) Two New Triterpene Glycosides from the Vietnamese Sea Cucumber Holothuria scabra

Han H, Xu QZ, Yi YH, Gong W, Jiao BH (2010) Two new cytotoxic disulfated holostane glycosides from the sea cucumber Pentacta quadrangularis. Chem Biodivers 7:158–167 . doi: 10.1002/cbdv.200800324

Han H, Yi YH, Li L, Liu BS, La MP, Zhang HW (2009) Antifungal active triterpene glycosides from sea cucumber Holothuria scabra CA152:128531. Yaoxue Xuebao 44:620–624

Kalinin VI, Avilov SA, Kalinovskii AI, Stonik VA, Branch F, Academy R (1992) Cucumarioside G3 - A minor triterpene glycoside from the holothurian Eupentacta fraudatrix. Chem Nat Compd 28:635–636

Kitagawa I, Akutsu H, Kyogoku Y, Zubrica H (1978a) Structure of Holothurin B. A pharmacologically

active triterpene oligoglycoside from the sea cucumber Holothuria leucospilota. Tetrahedron Lett 985–988

Kitagawa I, Inamoto T, Fuchida M, Okada S, Kobayashi M, Nishino T, Kyoboku Y (1980) Structures of Echinoside A and B, two antifungal oligoglycosides from the sea cucumber Actinopyga echinites (JAEGER). Chem Pharm Bull 28:1651–1653 . doi: 10.1248/cpb.37.3229

Kitagawa I, Kobayashi M, Hori M, Kyogoku Y (1989a) Marine Natural Producs. XVIII. Four lanostane-type triterpene oligoglycosides, bivittosides A,B,C, and D from the Okinawan sea cucumber Bohadschia bivittata (Mitsukuri). Chem Pharm Bull 37:61–67

Kitagawa I, Kobayashi M, Hori M, Kyogoku Y (1981a) Structures of four new triterpenoidal oligoglycosides, Bivittoside A, B, C, and D, from the sea cucumber Bohadschia bivittata MITSUKURI. Chem Pharm Bull 29:282–285 . doi: 10.1093/jxb/erl177

Kitagawa I, Kobayashi M, Inamoto T, Yasuzawa T, Kyogoku Y (1981b) The structure of six antifungal oligoglycosides, Stichlorosides A1,A2,Ba,B2,C, and C2, from the sea cucumber Stichopus chloronotus (Brandt). Chem Pharm Bull 29:2387–2391

Kitagawa I, Kobayashi M, Kyogoku Y (1982) Marine natural products. IX. Structural elucidation of triterpenenoidal oligoglycosides from the Bahamean sea cucumber Actinopyga agassizi Selenka. Chemi Pharm Bull 30:2045–2050

Kitagawa I, Kobayashi M, Son BW, Suzuki S, Kyogoku Y (1989b) Marine natural products. XIX. Pervicosides A, B, and C, lanostane-type triterpene oligoglycoside sulfates from the sea cucumber Holothuria pervicax. ChemiPharm Bull 37:1230–1234

Kitagawa I, Nishino T, Kyogoku Y (1979) Structure of holothurin A a biologically active triterpene-oligoglycoside from the sea cucumber holothuria leucospilota brandt. Tetrahedron Lett 1419–1422 . doi: 10.1016/S0040-4039(01)86166-9

Kitagawa I, Yamanaka H, Kobayashi M, Nishino T, Yosioka I, Sugawara T (1978b) Saponin and sapogenol. XXVII. Revised structures of holotoxins A & B, two antifungal oligoglycosides from the sea cucumber Stichopus japonicus Selenka. Chem Pharm Bull 26:3722–3731

Kobayashi M, Hori M, Kan K, Yasuzawa T, Matsu M, Suzuki S, Kitagawa I (1991) Marine Natural Products. XXVII Distribution of Lanostane-type triterpene oligoglycosides in ten kind of Okinawan sea cucumbers. Chem Pharm Bull 39:2282–2287 . doi: 10.1248/cpb.37.3229

Kokai Tokkyo Koho J (1984) Production of antibiotic perivicosides (Japanese patent, CA102:182848n)

Lakshmi V, Saxena A, Mishra SK, Raghubir R, Srivastava MN, Jain RK, Maikhuri JP, Gupta G (2008) Spermicidal Activity of Bivittoside D from Bohadschia vitiensis. Arch Med Res 39:631–638 . doi: https://doi.org/10.1016/j.arcmed.2008.06.007

Lakshmi V, Srivastava S, Mishra SK, Shukla PK (2012) Antifungal activity of bivittoside-D from Bohadschia vitiensis (Semper). Nat Prod Res 26:913–918 . doi: 10.1080/14786419.2010.534096

Liu B, Yi Y-H, Li L, Sun P, Han H, Sun GQ, Wang X, Wang Z-L (2008a) Argusides D and E, two new cytotoxic triterpene glycosides from the sea cucumber Bohadschia argus. Chem Biodivers 5:1425–1433

Liu BS, Yi YH, Li L, Sun P, Yuan WH, Sun GQ, Han H, Xue M (2008b) Argusides D and E, two new cytotoxic triterpene glycosides from the sea cucumber Bohadschia argus Jaeger. Chem Biodivers 5:1425–1433 . doi: 10.1002/cbdv.200890115

Liu BS, Yi YH, Li L, Sun P, Yuan WH, Sun GQ, Han H, Xue M, Sun GQ, Wang XH, Wang ZL (2008c) Argusides B and C, two new cytotoxic triterpene glycosides from the sea cucumber Bohadschia argus Jaeger. Chem Biodivers 5:1288–1297 . doi: 10.1002/cbdv.200890115

Liu BS, Yi YH, Li L, Zhang SL, Han H, Weng YY, Pan MX (2007) Arguside A: A new cytotoxic triterpene glycoside from the sea cucumber Bohadschia argus jaeger. Chem Biodivers 4:2845–2851 . doi: 10.1002/cbdv.200790234

Miyamoto T, Togawa K, Higuchi R, Komori T, Sasaki T (1990) Constituents of holothuroidea, II. Six newly identified biologically active triterpenoid glycoside sulfates from the sea cucumber Cucumaria echinata. Liebigs Ann der Chemie 453–460 . doi: 10.1002/jlac.199019900186

Oleinikova GK, Kuznetsova TA, Rovnykh N V, Kalinovskii AI, Elyakov GB (1982) Glycosides of marine

invertebrates. XVIII. Holothurin A2 from the Caribbean holothurian Holothuria floridana. Khim Prir Soedin 527–528

Rodriguez J, Castro R, Riguera R (1991) Holothurinosides: New antitumor non sulphated triterpenoid glycosides from the sea cucumber Holothuria forskali. Tetrahedron 47:4753–4762

Rodriguez J, Riguera R (1989) Lefevreiosides: four novel triterpenoid glycosides from the sea cucumber Cucumaria lefevrei. ChemInform 21: . doi: 10.1002/chin.199014299

Silchenko AS, Avilov SA, Antonov AS, Kalinovsky AI, Dmitrenok PS, Kalinin VI, Woodward C, Collin PD (2005a) Glycosides from the sea cucumber Cucumaria frondosa. IV. Structure of frondosides A2-4, A2-7, and A2-8, three new minor monosulfated triterpene glycosides. Can J Chem 83:2120–2126 . doi: 10.1139/v05-243

Silchenko AS, Kalinovsky AI, Avilov SA, Andryjaschenko P V., Dmitrenok PS, Kalinin VI, Martyyas EA, Minin K V. (2016) Fallaxosides C1, C2, D1 and D2, Unusual Oligosulfated Triterpene Glycosides from the Sea Cucumber Cucumaria fallax (Cucumariidae, Dendrochirotida, Holothurioidea) and Taxonomic Status of this Animal. Nat Prod Commun 11:939–945 . doi: 10.1177/1934578x1601100718

Silchenko AS, Kalinovsky AI, Avilov SA, Andryjaschenko P V., Dmitrenok PS, Kalinin VI, Yurchenko EA, Dautov S (2014) Structures of Violaceusosides C, D, E and G, Sulfated Triterpene Glycosides from the Sea Cucumber Pseudocolochirus violaceus (Cucumariidae, Dendrochirotida). Nat Prod Rep 9:391–399

Silchenko AS, Kalinovsky AI, Avilov SA, Andryjaschenko P V., Dmitrenok PS, Kalinin VI, Yurchenko EA, Dolmatov IY (2015) Colochirosides B1, B2, B3 and C, novel sulfated triterpene glycosides from the sea cucumber Colochirus robustus (Cucumariidae, Dendrochirotida). NPC Nat Prod Commun 10:1687–1694 . doi: 10.1080/13531040802284544

Silchenko AS, Kalinovsky AI, Avilov SA, Andryjaschenko P V., Dmitrenok PS, Martyyas EA, Kalinin VI (2012) Triterpene Glycosides from the Sea Cucumber Eupentacta fraudatrix. Structure and Cytotoxic Action of Cucumariosides A2, A7, A9, A10, A11, A13 and A14, Seven New Minor Non-Sulfated Tetraosides and an Aglycone with an Uncommon 18-Hydroxy Group. Nat Prod Commun Triterpene 7:845–852

Silchenko AS, Kalinovsky AI, Avilov SA, Kalinin VI, Andrijaschenko P V., Dmitrenok PS, Chingizova EA, Ermakova SP, Malyarenko OS, Dautova TN (2017) Nine new triterpene glycosides, magnumosides A1–A4, B1, B2, C1, C2and C4, from the Vietnamese sea cucumber Neothyonidium (=Massinium) magnum: Structures and activities against tumor cells independently and in synergy with radioactive irradiation. Mar Drugs 15:1–22 . doi: 10.3390/md15080256

Silchenko AS, Stonik VA, Avilov SA, Kalinin VI, Kalinovsky AI, Zakharenko AM, Smirnov AV, Mollo E, Cimino G (2005b) Holothurins B2, B3, and B4, new triterpene glycosides from Mediterranean sea cucumbers of the genus Holothuria. J Nat Prod 68:564–567 . doi: 10.1021/np049631n

Stonik VA, Mal'tsev II, Kalinovsky AI, Conde C, Elyakov GB (1983) Glycosides of marine invertebrates. XI Two new triterpene glycosides from holothurians of the family Stichopodidae. Plenum Publ Corp 177–181

Sun G, Li L, Yi Y, Yuan W, Liu B, Weng Y, Zhang SL, Wang ZL (2008) Two New cytotoxic nonsulfated pentasaccharide holostane (¼ 20-Hydroxylanostan-18-oic Acid g -Lactone) glycosides from the sea cucumber Holothuria grisea. Helv Chim Acta 91:1453–1460

Sun P, Liu BS, Yi YH, Li L, Gui M, Tang HF, Zhang DZ, Zhang SL (2007) A new cytotoxic lanostane-type triterpene glycoside from the sea cucumber Holothuria impatiens. Chem Biodivers 4:450–457 . doi: 10.1002/cbdv.200790037

van Dyck S, Flammang P, Meriaux C, Bonnel D, Salzet M, Fournier I, Wisztorski M (2010) Localization of secondary metabolites in marine invertebrates: Contribution of MALDI MSI for the study of saponins in Cuvierian tubules of H. forskali. PLoS One 5:e13923 . doi: 10.1371/journal.pone.0013923

Wu J, Yi Y-H, Tang H-F, Wu H-M, Zou Z-R, Lin H-W (2006a) Nobilisides A - C, Three New Triterpene

Glycosides from the Sea Cucumber Holothuria nobilis. Planta Med 72:932–935 . doi: 10.1055/s-2006-931603

Wu J, Yi YH, Tang HF, Zou ZR, Wu HM (2006b) Structure and cytotoxicity of new lanostane-type triterpene glycoside from the sea cucumber Holothuria hilla. Chem Biodivers 3:1249–1254 . doi: 10.1002/cbdv.200690126

Wu JUN, Yi Y-H, Tang H-F, Wu H-M, Zhou Z-R (2007) Hillasides A and B, two new cytotoxic triterpene glycosides from the sea cucumber Holothuria hilla Lesson. J Asian Nat Prod Res 9:609–615 . doi: 10.1080/10286020600882676

Yuan WH, Yi YH, Tan RX, Wang ZL, Sun GQ, Xue M, Zhang HW, Tang HF (2009a) Antifungal triterpene glycosides from the sea cucumber Holothuria (Microthele) axiloga. Planta Med 75:647–653 . doi: 10.1055/s-0029-1185381

Yuan WH, Yi YH, Tang HF, Liu BS, Wang ZL, Sun GQ, Zhang W, Li L, Sun P (2009b) Antifungal triterpene glycosides from the sea cucumber Bohadschia marmorata. Planta Med 75:168–173 . doi: 10.1055/s-0028-1088348

Yuan WH, Yi YH, Tang HF, Liu BS, Wang ZL, Sun GQ, Zhang W, Li L, Sun P (2009c) Antifungal triterpene glycosides from the sea cucumber Bohadschia marmorata. Planta Med 75:168–173 . doi: 10.1055/s-0028-1088348

Zhang S-L, Li L, Sun P, Yi Y-H (2008) Lecanorosides A and B, two new triterpene glycosides from the sea cucumber Actinopyga lecanora. J Asian Nat Prod Res 10:1097–1103 . doi: 10.1080/10286020701604813

Zhang SY, Yi YH, Tang HF (2006a) Bioactive triterpene glycosides from the sea cucumber Holothuria fuscocinerea. J Nat Prod 69:1492–1495 . doi: 10.1021/np060106t

Zhang SY, Yi YH, Tang HF, Li L, Sun P, Wu J (2006b) Two new bioactive triterpene glycosides from the sea cucumber Pseudocolochirus violaceus. J Asian Nat Prod Res 8:1–8 . doi: 10.1080/10286020500034972

Zhang XM, Han LW, Sheng WL, Li X Bin, Zhang SS, Guo JL, Lin HW, Liu KC (2020) Two novel non-holostane type glycosides from the viscera of sea cucumber Apostichopus japonicus. J Asian Nat Prod Res 22:1–9 . doi: 10.1080/10286020.2019.1576643

Zhang XM, Li X Bin, Zhang SS, He QX, Hou HR, Dang L, Guo JL, Chen YF, Yu T, Peng DJ, Han LW, Liu KC (2018) LC-MS/MS Identification of Novel Saponins from the Viscera of Sea Cucumber Apostichopus japonicus. Chem Nat Compd 54:721–725 . doi: 10.1007/s10600-018-2454-4

Zhao Q, Liu ZD, Xue Y, Wang JF, Li H, Tang QJ, Wang YM, Dong P, Xue CH (2011) Ds-echinoside A, a new triterpene glycoside derived from sea cucumber, exhibits antimetastatic activity via the inhibition of NF-κB-dependent MMP-9 and VEGF expressions. J Zhejiang Univ Sci B 12:534–544 . doi: 10.1631/jzus.B1000217

Zou Z, Yi Y, Wu H, Yao X, Du L, Jiuhong W, Liaw CC, Lee KH (2005) Intercedensides D-I, cytotoxic triterpene glycosides from the sea cucumber Mensamaria intercedens lampert. J Nat Prod 68:540–546 . doi: 10.1021/np040205b

Zou ZR, Yi YH, Wu HM, Wu JH, Liaw CC, Lee KH (2003) Intercedensides A-C, three new cytotoxic triterpene glycosides from the sea cucumber Mensamaria intercedens Lampert. J Nat Prod 66:1055–1060 . doi: 10.1021/np030064y
